# Nonclinical safety assessment of SBT777101, a CAR-engineered regulatory T cell therapy for autoimmune diseases

**DOI:** 10.1093/toxsci/kfag125

**Published:** 2026-09-15

**Authors:** Herve Lebrec, Jonathan M Clingan, Anne-Renee van der Vuurst de Vries, Lauren Mihalcik, Yuchi Honaker, Sara Charmsaz, Seonhwa Dura, Kathryn A Hooper, Yuanyuan Xiao, Michelle Blake, Max Fiebiger, Radha Dhayal, Christine Saechao, Alexandre Iannello, Fred Ramsdell, Jeffrey A Bluestone, Josh Beilke

**Affiliations:** Sonoma Biotherapeutics, South San Francisco, CA 94080, United States; Sonoma Biotherapeutics, South San Francisco, CA 94080, United States; Sonoma Biotherapeutics, Seattle, WA 98119, United States; Aclairo Pharmaceutical Development Group, Vienna, VA 22182, United States; Sonoma Biotherapeutics, Seattle, WA 98119, United States; Sonoma Biotherapeutics, Seattle, WA 98119, United States; Sonoma Biotherapeutics, Seattle, WA 98119, United States; Sonoma Biotherapeutics, Seattle, WA 98119, United States; Sonoma Biotherapeutics, South San Francisco, CA 94080, United States; Sonoma Biotherapeutics, Seattle, WA 98119, United States; Sonoma Biotherapeutics, South San Francisco, CA 94080, United States; Sonoma Biotherapeutics, South San Francisco, CA 94080, United States; Sonoma Biotherapeutics, South San Francisco, CA 94080, United States; Sonoma Biotherapeutics, South San Francisco, CA 94080, United States; Sonoma Biotherapeutics, Seattle, WA 98119, United States; Sonoma Biotherapeutics, South San Francisco, CA 94080, United States; Sonoma Biotherapeutics, Seattle, WA 98119, United States

**Keywords:** regulatory T cells, chimeric antigen receptor, immunosuppression, new approach methodologies

## Abstract

Chimeric antigen receptor (CAR)-regulatory T cell (Treg) therapies represent a promising approach for autoimmune diseases, but their nonclinical safety assessment remains challenging due to antigen-, disease-, and species-specific pharmacology that limits the relevance of conventional animal models. SBT777101, a CAR-Treg therapy targeting citrullinated proteins associated with rheumatoid arthritis and hidradenitis suppurativa, was evaluated using a nonclinical strategy centered on in vitro and ex vivo New Approach Methodologies (NAMs) and in vivo studies benchmarked against untransduced polyclonal Tregs, which are manufactured identically but lack the CAR and have an established clinical safety record, complemented by immunodeficient mouse studies. We hypothesized that CAR engineering would not destabilize the Treg phenotype or introduce new toxicological liabilities relative to polyclonal Tregs. SBT777101 maintained phenotypic stability under repeated stimulation and proinflammatory conditions, produced minimal proinflammatory cytokines following activation, and demonstrated immunomodulatory activity comparable to untransduced Tregs. Tissue cross-reactivity and primary cell studies identified limited membrane-associated binding, no evidence of CAR-mediated cytotoxicity or activation in normal human tissues, and no off-target binding by broad protein array screening. Immunosuppressive activity toward natural killer and CD8^+^ T cell responses was limited and comparable to polyclonal Tregs. Lentiviral integration site analyses demonstrated polyclonal integration profiles without evidence of transformation or abnormal growth in a cytokine- and activation-independent growth assay. This study establishes a combined in vivo and NAM-based nonclinical safety framework for CAR-Treg therapies and demonstrates that CAR engineering of SBT777101 does not introduce new safety liabilities relative to unmodified polyclonal Tregs. These findings supported progression of SBT777101 into Phase 1 clinical trials and offer a generalizable approach for evaluating the safety of future CAR-Treg therapeutics.

SBT777101 is an ex vivo expanded, cryopreserved, autologous CD4^+^CD127^lo/−^CD25^+^ regulatory T cell (Treg) preparation. It is transduced with a lentiviral vector encoding a chimeric antigen receptor (CAR) specific for immunodominant regions of posttranslationally modified citrullinated proteins (CitP), along with an inert truncated epidermal growth factor receptor (EGFRt) tag used for identification and isolation of modified Tregs. Upon antigen recognition, SBT777101 is hypothesized to act through direct and local bystander suppression of inflammation. Tregs traffic inherently to inflamed tissues (Campbell 2015); by using a CitP-specific CAR that bypasses the need for major histocompatibility complex (MHC)-restricted antigen presentation, SBT777101 is designed to expand locally and restore immune balance.

The anti-inflammatory effects of SBT777101 are mediated by well-characterized Treg suppressive mechanisms (Bluestone and Anderson 2020), including immunosuppressive cytokine production (e.g. interleukin (IL)-10) (Rubtsov et al. 2008), inhibitory checkpoint receptor expression (e.g. cytotoxic T-lymphocyte-associated antigen 4 [CTLA4]) (Takahashi et al. 2000), and high CD25 expression, which acts as an IL-2 “sink” to starve effector T cells (Teff) (Pandiyan et al. 2007). Collectively, these mechanisms are expected to reduce the number and function of inflammatory cells. Tregs can also produce amphiregulin to support tissue repair, further extending CAR-Treg activity (Arpaia et al. 2015; Lei et al. 2015).

The recognition domain of SBT777101 was derived from an autoantibody isolated from a rheumatoid arthritis (RA) patient (Steen et al. 2019; Sahlstrom et al. 2020). The CAR was selected for its specificity to CitP over noncitrullinated proteins, with citrullinated vimentin (CV) identified as a predominant binder. While citrullination is a normal physiological process, it can become dysregulated in autoimmune diseases such as RA (Darrah and Andrade 2018) or hidradenitis suppurativa (HS) (Byrd et al. 2019), generating neoantigens and anticitrullinated protein antibodies (ACPAs) that drive further autoimmune responses and exacerbate pathogenesis (England et al. 2017). We hypothesized that this CAR would enable directed Treg activation across a high percentage of RA or HS patients based on in vitro responses to disease-associated CitP.

Compared with polyclonal approaches, antigen-specific Treg therapy offers enhanced antigen- and disease-specific activity but raises unique “on-target, off-disease” safety considerations. Given the limitations of evaluating human cells in animals, SBT777101’s nonclinical safety evaluation combined in vitro “new approach methodologies” (NAMs) with in vivo assessments. Leveraging in vitro assays, tissue-mapping technologies, and genomic analysis, this approach offers a robust template for characterizing therapeutic cell products in a manner directly relevant to human physiology. The toxicology program addressed five critical safety domains: Target specificity, lineage stability, lack of cytotoxicity, absence of systemic immunosuppression, and lack of abnormal cell growth upon vector integration. Results were consistent with observations from targeted studies conducted in immunodeficient mice. In vitro and ex vivo NAMs directly addressed target specificity, functional lineage stability, cytotoxicity, and immunosuppression risk using human cells, while in vivo studies in immunodeficient mice, conducted after Health Authorities’ feedback, focused on understanding SBT777101’s impact on tissues naturally expressing CitP, off-disease activation risk, and phenotype stability under active inflammatory challenge.

The first phase of characterization mapped SBT777101 CAR binding to define potential on-target, off-disease activity. Beyond confirming specific CitP recognition with no detectable cross-reactivity to noncitrullinated counterparts or off-target proteins, this mapping established a baseline for target expression in healthy tissues. We also assessed the functional consequences of this binding to confirm SBT777101 lacks cytotoxicity against human cells across various cell types and organ systems.

Beyond target recognition, we subjected SBT777101 to functional “stress tests” across a broad spectrum of physiological conditions. These assessments aimed to define the safety boundaries of CAR-mediated activation under pressure by evaluating Treg phenotype stability during repeated stimulation under proinflammatory conditions both in vitro and in a mouse graft-versus-host disease (GvHD) model, and by characterizing the cytokine profile of SBT777101 relative to that of expanded Teff to assess cytokine release syndrome (CRS) risk.

Because CitP occurs naturally in many tissues (Baka et al. 2012), we evaluated the risk of broad, off-disease immunosuppression by testing whether SBT777101 activation suppresses cancer immune surveillance and infection-fighting functions of NK and cytotoxic CD8^+^ T cells, and by investigating CitP expression in malignant and infectious tissues.

Lastly, we addressed the genomic consequences of lentiviral engineering. Through high-resolution insertional site analysis and cytokine-independent proliferation assays, we sought to confirm that the modification of Tregs does not lead to clonal dominance or autonomous, factor-independent cell growth. Collectively, these molecular and functional assessments provide a high-resolution safety profile directly relevant to human physiology, establishing a robust paradigm for nonclinical evaluation of engineered Treg therapies.

## Materials and methods

### CAR-Jurkat reporter assay

NFAT-luciferase reporter Jurkat cells (BPS Bioscience) were stably transduced with the SBT777101 CAR. A panel of 13 CitP known to be citrullinated in inflammatory disease and their noncitrullinated counterparts (Cayman Chemical) was used to coat 96-well plates at an initial concentration of 50 µg/ml, followed by 3-fold serial dilutions. CAR-Jurkat cells (5 × 10^4^ cells/well; 3 technical replicates) were added to the plates and incubated for 20 to 24 h at 37°C. Following incubation, Bio-Glo substrate (Promega) was added, and luminescence was measured using a SpectraMax plate reader. Background signal was established using media-only controls. CitP were enzymatically citrullinated recombinant proteins, distinct from synthetic peptides or patient-derived material.

### Generation of SBT777101 Tregs

Human CD4^+^CD25^+^ cells were isolated from healthy donor, RA patient, or HS patient primary sources using immunomagnetic enrichment, followed by fluorescence-activated cell sorting for CD4^+^CD25^+^CD127^lo^ Tregs. Resulting cells were activated using anti-CD3/CD28-coated beads and in the presence of exogenous human IL-2. Cells were transduced on Day 3 with a lentiviral vector encoding the SBT777101 CAR and EGFRt. Tregs were expanded until Day 14, at which point cells were harvested, de-beaded, and either used fresh or cryopreserved (Stoops et al. 2025).

### Primary Treg proliferation assay

96-Well plates were coated with a titration of citrullinated proteins (starting at 50 µg/ml). Cryopreserved SBT777101 Tregs were thawed and rested for 3 d in RPMI medium supplemented with 300 U/ml human IL-2. Following the rest period, cells were labeled with carboxyfluorescein succinimidyl ester (CFSE; Thermo Fisher) and added to the antigen-coated plates (5 × 10^4^ cells/well; 3 technical replicates). After 72 h of culture, cell proliferation was assessed by CFSE dilution using flow cytometry. Proliferation was quantified as the percentage of CFSE^lo^ cells within the CD4^+^EGFR^+^ population.

### Off-target binding screen (Retrogenix)

To evaluate potential off-target binding, the SBT777101 CAR was screened against a library of HEK293 cells expressing 5828 noncitrullinated full-length human plasma membrane proteins and cell surface-tethered human-secreted proteins, plus 398 human heterodimers (Retrogenix® Cell Microarray; Charles River Laboratories). Separately, CV and citrullinated fibrinogen were tested for staining as positive controls; noncitrullinated vimentin served as a negative control. Briefly, a human Fc fusion protein consisting of the SBT777101 CAR scFv fused to a human IgG1-Fc (7101-hIgG1-Fc) was applied to the microarray. Binding was detected via immunofluorescence using an Alexa Fluor 647 labeled antihuman IgG Fc detection antibody. Potential hits in the library screen were validated in secondary confirmatory assays using live or fixed HEK293 cells, and by flow cytometry.

### Immunohistochemistry analysis

The SBT777101 CAR fused to mouse IgG2a-Fc (7101-mIgG2a-Fc) was applied to a library of cryosections of normal human tissues (at least 3 donors per tissue, where available), NOD-*Prkdc^em26Cd52^Il2rg^em26Cd22^*/NjuCrl (NCG; Charles River Laboratories) mouse tissues (*n* = 15 mice) and a panel of malignant and infectious disease-derived tissues. Human sections were evaluated at concentrations of 2 and 5 µg/ml 7101-mIgG2a-Fc, while mouse sections were evaluated at 10 µg/ml; bound 7101-mIgG2a-Fc was detected using Dako EnVision + peroxidase-labeled antimouse IgG polymer (Agilent/Dako) for 30 min, followed by Dako DAB+ substrate-chromogen solution for 8 min. Positive controls included the neuroblastoma cell line SKNBE2 overexpressing peptidylarginine deaminase 2 (PAD2) or 4 (PAD4) as well as UV-resin spot slides containing citrullinated fibrinogen or vimentin. Negative controls included an isotype-matched mIgG2a-Fc protein, wild-type SKNBE2 cells, and noncitrullinated vimentin UV-resin spot slides. Staining was evaluated by a board-certified pathologist and scored for intensity (0 to 4+) and frequency (0% to 100%).

### Specificity and toxicity against primary human cells

Primary human cells (mammary epithelial cells [HMEC; Lonza Cat#CC-2551], normal bronchial epithelial cells [NHBE; Lonza Cat#CC2451], renal proximal tubule epithelial cells [RPTEC; Lonza Cat#CC2553], prostate epithelial cells [PrEC; Lonza Cat#CC2555], hepatocytes [Lonza Cat#HUCPG], brain astrocytes [HBA; Neuromics Cat#HMP202], iPSC-derived myocytes [Tempo Bioscience Cat#1014], iPSC-derived cortical neurons [Tempo Bioscience Cat#201], and monocyte-derived macrophages [internally derived]) and the positive-control SNU449 cell line (ATCC Cat#CRL-2234) were assessed for binding of the SBT777101 scFv by flow cytometry. Cells were stained for surface binding (7101-mIgG2a-Fc) or intracellularly (7101-hIgG1-Fc) following fixation and permeabilization, followed by incubation with F(ab’)2-Goat anti-Mouse IgG APC (eBioscience Cat#50-112-9759) or Goat anti-Human IgG Fc PE (eBioscience Cat#21-4998-82).

Target cells were plated in 96-well plates in triplicate and cultured 1:1 with untransduced (UT) or SBT777101 Tregs (and CV-beads in SNU449 assays). Following 20 to 24 h of incubation, the Tregs were harvested and analyzed for expression of activation markers CD69 (ThermoFisher Cat#78-0047-42) and CD71 (ThermoFisher Cat#47-0719-41) by flow cytometry. To assess potential off-target cytotoxicity, the viability of the adherent primary human cells was measured using the CellTiter-Glo Luminescent Cell Viability Assay (Promega) according to the manufacturer’s instructions. The percentage of remaining cells was expressed as a percentage of the mean Relative Light Units of the “no T cell” treatment control wells.

### Single-dose toxicity study in NCG mice

Animal care and use were in accordance with the Institutional Animal Care and Use Committee (IACUC) at Explora BioLabs (an AAALAC-accredited facility). Forty male and 40 female NCG mice (Charles River Laboratories) aged 8 wk were housed under pathogen-free conditions with *ad libitum* access to food and water. Mice (*n* = 20/sex/group) were randomized into treatment cohorts, with each group subdivided into two sets (*n* = 10/sex/set) for terminal analysis at Day 7 or Day 14 postdose.

Groups 1 and 2 were administered cryopreservation media (vehicle) diluted in saline. Group 3 received 5 × 10^6^ CAR^−^EGFR^+^ Tregs per animal, and Group 4 received 5 × 10^6^ SBT777101 Tregs per animal. Mice were administered cells via intravenous (IV) injection. The dose was selected to represent a multiple of the proposed clinical dose when converted to a cells/kg basis. CAR^−^EGFR^+^ Tregs were generated using the same donor and process as for SBT777101, except that they were transduced with a vector that contained the EGFRt sequence but no CAR sequence. Mice in groups 2 through 4 also received 160,000 U of human IL-2 to promote Treg survival.

Following euthanasia on Day 7 or 14, mice were subjected to gross necropsy and tissues were collected for organ weights and histopathology. Transplanted cells were quantified in blood and select tissues, and phenotyped in blood.

### Digital droplet polymerase chain reaction

Genomic DNA was extracted from blood and snap-frozen tissue samples using the DNeasy Blood & Tissue 96-well plate kit (Qiagen) according to the manufacturer’s instructions. Digital droplet polymerase chain reaction (ddPCR) was performed on a QX200 system (Bio-Rad) using primers and probes targeting the RRE region of the lentiviral vector. Each reaction was performed in a total volume of 22 μl with 25 ng of DNA input. Thermal cycling conditions were as follows: 95°C for 10 min; 40 cycles of 95°C for 30 s and 60°C for 60 s (ramp rate 2°C/s); 98°C for 10 min; hold at 4°C. CAR vector copy number was expressed as RRE copies per reaction. The limit of blank and lower limit of quantitation were determined as part of the assay qualification.

### In vitro *Treg stability under inflammatory conditions*

Following manufacturing, SBT777101 cells generated from 3 healthy donors were rested for 3 d in culture media supplemented with 300 U/ml IL-2. On Day 0, cells were stimulated with either anti-CD3/CD28-coated beads or CV-beads. Cultures were maintained in either standard Treg media (IL-2 alone) or proinflammatory media supplemented with IL-2 and a cytokine cocktail formulated to reflect the cytokine milieu of RA synovial fluid, based on concentrations determined from an internal analysis of patient samples and consistent with published reports (Gomez-Puerta et al. 2013; Koo et al. 2020; Ji et al. 2021; Skrzypkowska et al. 2022), including TNF-α, IL-6, IL-23, IL-1β, and IL-18. Cells were restimulated weekly (Days 0, 7, 14, and 21) with the respective bead types. FOXP3 expression was monitored longitudinally via flow cytometry (ThermoFisher Cat#53-4776-42) on Days 0, 7, 14, 21, and 28.

### Xenogeneic GvHD model

Animal care and use were in accordance with the Institutional Animal Care and Use Committee (IACUC) at Explora BioLabs (an AAALAC-accredited facility). Eighty female NCG mice (Charles River Laboratories) aged 12 wk were housed under pathogen-free conditions with *ad libitum* access to food and water. Mice (*n* = 10/group) were randomized into treatment cohorts, with each group subdivided into two sets (*n* = 5/set) for distinct terminal analyses (Set A: Histopathology; Set B: Cytokine and Treg Stability).

On Day 1, mice received 2.0 Gy of sublethal irradiation. Four hours postirradiation, animals received intranasal saline or LPS (10 µl at 5 mg/kg). LPS challenges were repeated 4 h prior to PBMC infusion on Day 2 and again on Day 7. GvHD was induced on Day 2 via IV infusion of 5.0 × 10^6^ human PBMCs (Groups 3 to 8). On Day 3, mice received an IV infusion of vehicle (Groups 1 to 4), 5.0 × 10^6^ CAR^−^EGFR^+^ Tregs (Groups 5 to 6), or 5.0 × 10^6^ SBT777101 Tregs (Groups 7 to 8); CAR^−^EGFR^+^ Tregs and SBT777101 Tregs were derived from the same donor, with the dose selected to represent a multiple of the proposed clinical dose when converted to a cells/kg basis. Mortality, moribundity, body weight, and clinical scores were assessed daily.

Animals were euthanized and subjected to necropsy on Day 13 (Set B) or Day 14 (Set A). Tissues for histopathology were preserved in 10% neutral-buffered formalin and assessed by a board-certified pathologist. For Set B, serum was isolated from whole blood to quantify circulating human cytokines using the Meso Scale Discovery (MSD) platform. Spleens were dissociated into single-cell suspensions for phenotypic analysis by flow cytometry; FOXP3 expression was analyzed by flow cytometry specifically within the EGFR^+^ (transduced) cell population.

### *Analysis of SBT777101* in vitro *cytokine production*

SBT777101 Tregs and expanded UT Teff were generated from healthy donors and patients with rheumatoid arthritis (RA) or hidradenitis suppurativa (HS). Cells were stimulated for 24 h with either anti-CD3/CD28 beads or CV-coated beads (SBT777101 only). Cell-free supernatants were harvested and stored at −80°C until analysis. Concentrations of granulocyte-macrophage colony-stimulating factor (GM-CSF), tumor necrosis factor (TNF)-α, interferon (IFN)-γ, IL-17, and IL-10 were quantified using a Luminex multiplex bead-based assay.

### NK cell suppression assay

Natural killer (NK) cells were enriched from healthy donor PBMCs using a negative selection immunomagnetic kit (Miltenyi Biotec) and rested overnight in media supplemented with 100 U/ml IL-2. Target K562 cells were labeled with CFSE (Thermo Fisher) prior to co-culture.

Autologous UT or SBT777101 Tregs were thawed and enriched for CAR expression via EGFR-PE staining followed by isolation using anti-PE immunomagnetic beads (Miltenyi Biotec). UT or SBT777101 Tregs were then cultured for 72 h under three conditions: Unstimulated, TCR-stimulated (anti-CD3/CD28 beads), or CAR-stimulated (CV-beads; SBT777101 only).

NK cells and CFSE-labeled K562 cells were combined at a 2:1 NK-to-K562 ratio, in 3 technical replicates for each donor. UT or SBT777101 Tregs were added at a 1:1 Treg-to-NK ratio under 4 distinct functional conditions: (i) resting, (ii) TCR preactivated, (iii) CAR preactivated (SBT777101 only), or (iv) resting with concomitant CAR activation (addition of CV-beads at the time of co-culture). After 4 h, K562 viability was assessed by 7-AAD staining of the CFSE^+^ population via flow cytometry and reported as the percentage of 7-AAD^+^ cells of K562 cells.

### CD8^±^ T cell suppression assay

Monocytes and CD8^+^ T cells were isolated from healthy donor leukopaks using immunomagnetic enrichment and cryopreserved. Immunocult™ Dendritic Cell Culture Kit (StemCell Technologies) was used according to manufacturer’s instructions to generate dendritic cells (DCs) and cells were harvested on Day 6 for use as antigen-presenting cells (APCs).

Autologous UT or SBT777101 Tregs were thawed and enriched for CAR expression via EGFR-PE staining followed by isolation with anti-PE immunomagnetic beads (Miltenyi Biotec). UT or SBT777101 Tregs were then cultured for 72 h under three conditions: Unstimulated, TCR-stimulated (anti-CD3/CD28 beads), or CAR-stimulated (CV-beads; SBT777101 only).

To assess suppressive activity, autologous monocyte-derived DCs were pulsed with CERI peptides (MHC-I-restricted epitopes from Cytomegalovirus, Epstein Barr Virus, Human Respiratory Syncytial Virus, and Influenza A Virus) for 1 h, then combined with autologous CD8^+^ T cells (1:5 DC-to-CD8^+^ T cell ratio) to initiate an antigen-specific recall response. UT or SBT777101 Tregs were added at a 1:10 Treg-to-CD8^+^ T cell ratio under 4 distinct functional conditions: (i) resting, (ii) TCR preactivated, (iii) CAR preactivated (SBT777101 only), or (iv) resting with concomitant CAR activation (addition of CV-beads at the time of co-culture). After 48 h of co-culture, supernatants were harvested and analyzed for IFN-γ concentration via MSD.

### Integration site analysis

Genomic DNA was extracted from SBT777101 derived from 4 donors and analyzed by Creative Biogene (Shirley, NY) using high-resolution Enrichment-Linked Linear Modeling PCR followed by Illumina MiSeq sequencing. Briefly, 1500 ng of DNA was sheared, linearly amplified with LTR-specific primers, and ligated to barcoded linkers before exponential PCR. Sequences were filtered (Phred ≥ 30) and aligned to the human genome (GRCh38.p13) via the GENE-IS suite. Clonal diversity was assessed using the Polyclonal-Monoclonal Distance (PMD) index, while common integration sites were defined as integration site (IS) clusters within 50 kbp. Finally, oncogenic risk was evaluated by cross-referencing IS against the Cancer Gene Census database (v90) within a 100 kbp window of known transcription start sites.

### Assessment of IL-2 independent growth

SBT777101 derived from three donors was cultured in the presence or absence of 300 U/ml of human IL-2, with cytokine replenished every 4 d. Proliferation and viability were monitored for 28 d, with measurements performed every 4 d using acridine orange (AO) and DAPI.

### Statistical analysis

Data were graphed using GraphPad Prism (version 9.3.1) or R (version 4.2.1); statistical analyses were done using GraphPad Prism. Comparisons between two groups were performed using a two-tailed unpaired *t*-test. Comparisons across three or more groups were performed using one-way or two-way ANOVA with Tukey’s or Sidak’s multiple comparisons test, respectively, or the Kruskal–Wallis test with Dunn’s multiple comparisons *post hoc* test, as appropriate for the experimental design. A *P*-value <0.05 was considered statistically significant. Biological replicates (independent donors) were treated as independent observations; technical replicates were averaged prior to statistical comparison. Specific statistical tests applied to each dataset are described in the corresponding figure legends.

## Results

### SBT777101 CAR specifically recognizes citrullinated proteins without off-target reactivity

To evaluate the specificity of the SBT777101 CAR, human Jurkat NFAT-luciferase reporter cells were stimulated with CitP associated with RA. Proteins that demonstrated activity in the Jurkat reporter assay were further evaluated for their potential to activate SBT777101 drug product (DP). In both SBT777101 CAR-expressing Jurkat reporter cells (Fig. 1) and SBT777101 DP (Fig. 2), potent activation was observed upon exposure to certain citrullinated proteins, including CV, glial fibrillary acidic protein (GFAP), and fibrinogen. However, this response was selective and activation was not observed in response to all citrullinated proteins. Additionally, no activation was observed in the presence of noncitrullinated proteins, demonstrating specificity for the citrullinated forms of antigens.

**Fig. 1.**
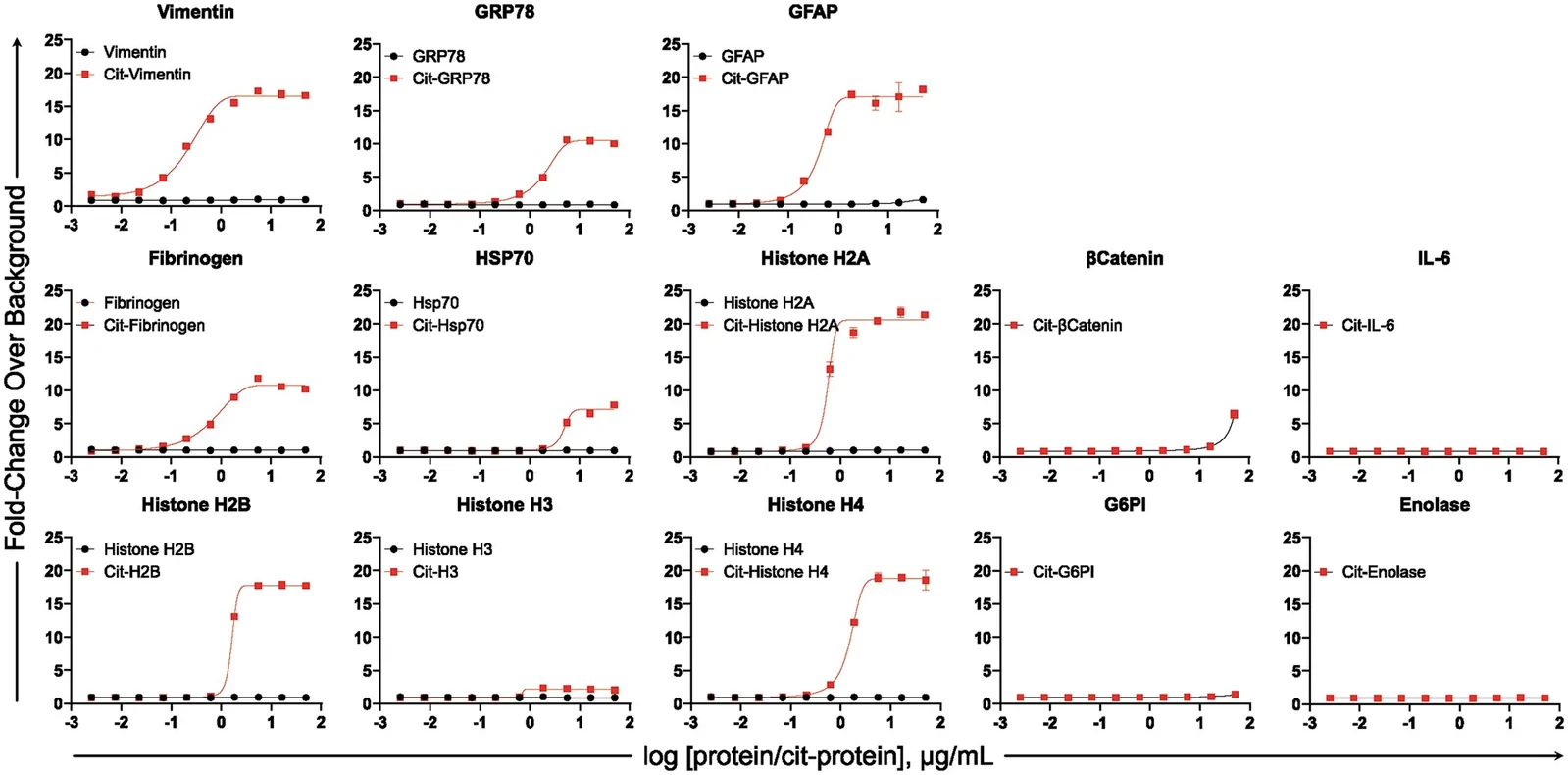
Selective activation of SBT777101 Jurkat reporter cells by citrullinated proteins. Representative dose–response curves showing fold-increase in luminescence (*y*-axis) relative to baseline following incubation with increasing concentrations (log scale, *x*-axis) of citrullinated (red) or noncitrullinated (black) protein targets. Data points represent the mean ± SD of 3 replicates.

**Fig. 2.**
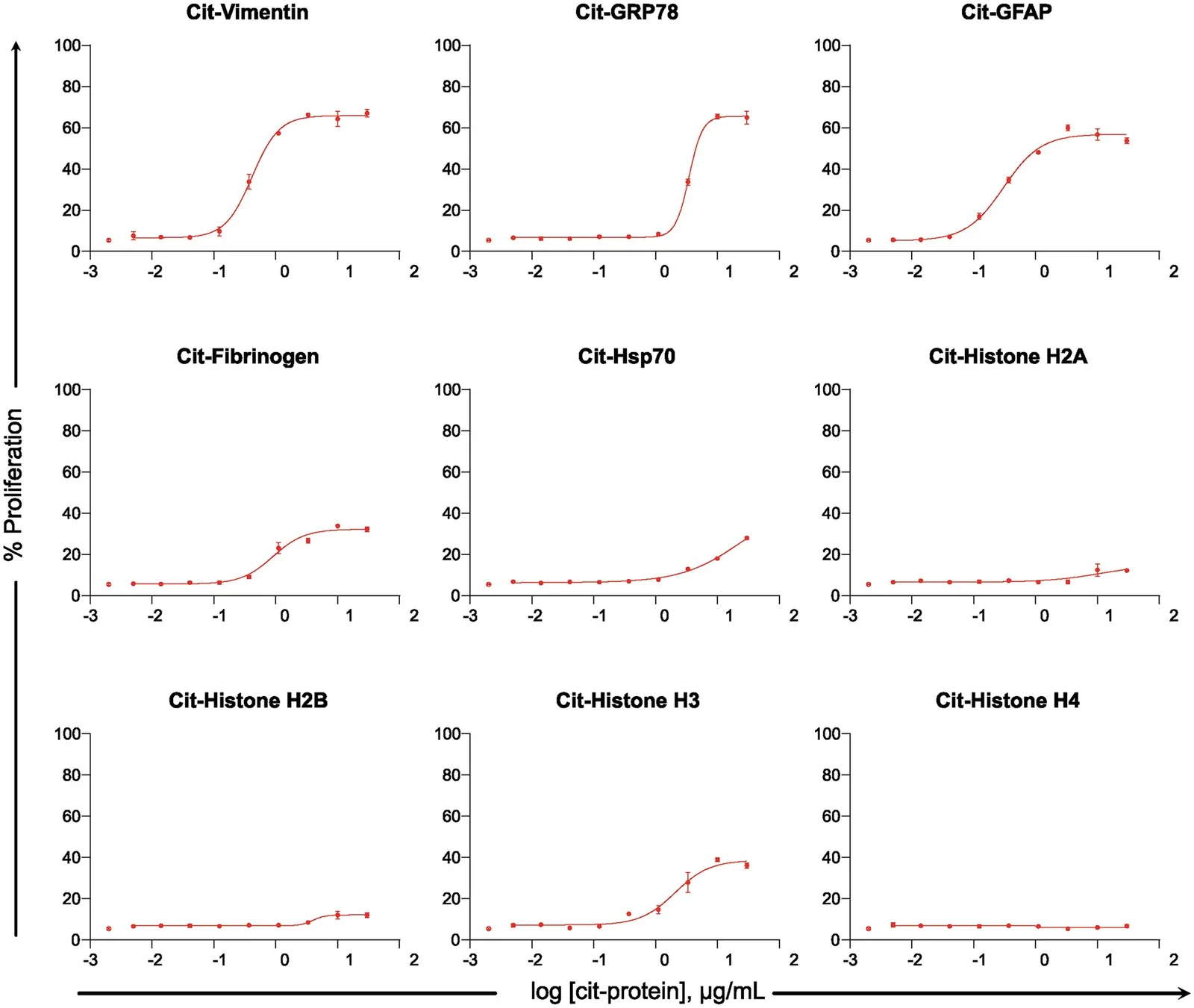
Proliferation of primary SBT777101 cells in response to citrullinated proteins. Primary SBT777101 CAR-Tregs were incubated with increasing concentrations (log scale, *x*-axis) of citrullinated protein targets. Cellular proliferation was measured by CFSE dilution at 72 h poststimulation. Data from a representative donor are shown; values represent mean ± SD of 3 technical replicates.

The Retrogenix™ Cell Microarray Technology was used to screen for specific off-target binding interactions of the SBT777101 CAR scFv, formatted as a human IgG1 Fc-fusion protein (7101-hIgG1-Fc), against a panel of human proteins. In a separate assay used to determine appropriate amounts of 7101-hIgG1-Fc to be used for the assay, binding to CV and Cit-fibrinogen was observed, while no binding was observed to noncitrullinated vimentin or with the isotype control, confirming the validity of conditions used for the screen. Off-target binding was detected against multiple Fc gamma receptors (FcγR) in both fixed and live cell microarrays: FCGR2B (isoforms 5 and IIB1), FCGR3A, and FCGR3B (Table S1). To assess the specificity of these interactions, binding was further assessed by flow cytometry. Strong binding was observed to the FcγR identified in the cell microarrays; however, strong binding was also observed with the SBT777101 CAR scFv formatted as a full-length human IgG1 antibody (7101-hIgG1) and a negative control hIgG1 antibody, while no binding was observed using the SBT777101 CAR binder formatted as a mouse IgG2a-Fc (7101-mIgG2a-Fc). These data indicate that the observed interactions of the SBT777101 CAR with FcγR were due to the human Fc domain of the 7101-hIgG1-Fc, and not the CAR binding domain. Because the SBT777101 CAR does not contain any Fc elements, this finding is not applicable to the SBT777101 DP.

### Mostly intracellular expression of citrullinated proteins in healthy human and mouse tissues

To evaluate the risk of on-target/off-disease reactivity, binding of 7101-mIgG2a-Fc was assessed across a panel of healthy human and NCG mouse tissues. In human tissues, membrane binding of the fusion protein was limited to epithelial cells in select tissues (esophagus, kidney, large intestine, liver, mammary gland, prostate, pituitary, and salivary gland) and mononuclear leukocytes in most tissues, with staining often consisting of cytoplasmic granules lining the cell surface rather than a distinct membrane border. This staining was primarily rare and observed in either 5% or less of all cells within the tissue (e.g. less than 5% of the tissue was composed of epithelial cells) or 5% or less of cells of that cell type (e.g. less than 5% of epithelial cells with membrane staining). Extracellular binding was observed in presumed glandular secretory or excretory material within the lumens of glands and kidney tubules, or within vessels, as well as many of the human tissues examined. This binding was often observed over stromal or interstitial tissue elements and could not be co-localized to a specific cell type or tissue structure. All other binding observed was cytoplasmic or nuclear in nature and considered consistent with the reported expression of the citrullinating enzymes PAD2 and PAD4 in various human tissues. A full summary of binding is provided in Table 1, with representative immunohistochemistry images provided in Fig. 3.

**Fig. 3.**
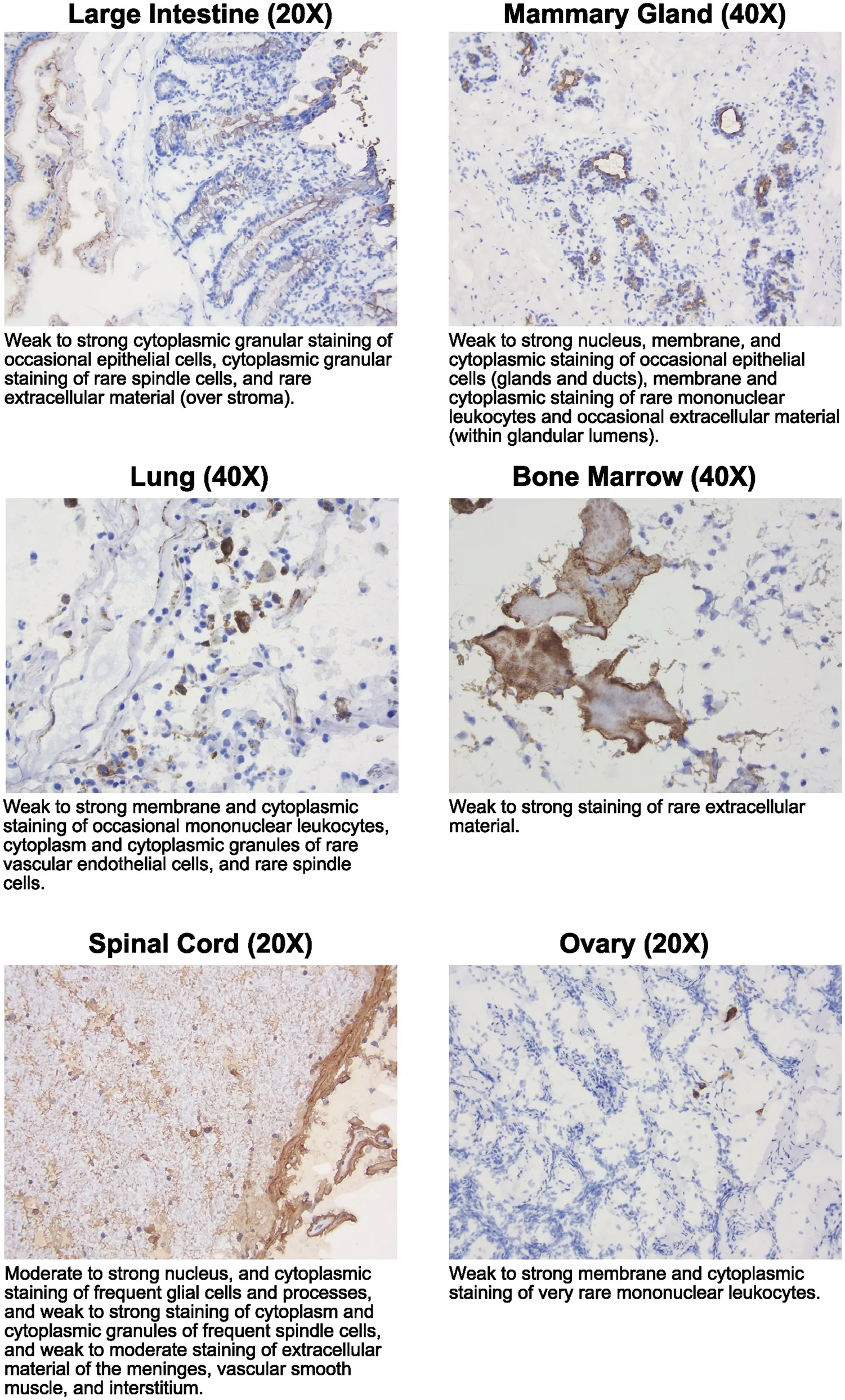
Representative immunohistochemical localization of SBT777101 scFv in normal human tissues. (A–F) Representative images demonstrate the range of observed staining intensities and cellular localization patterns following incubation with 5 μg/ml of scFv-mIgG2a. Qualitative binding profiles were characterized by a board-certified pathologist. Specific tissue descriptions and magnifications are indicated within each panel.

**Table 1.** Cross-reactivity profile of SBT777101 scFv in normal human tissues.

Cellular compartment	Cell type	Tissue
**Membrane and cytoplasmic localization, with nuclear staining in some tissues**	Epithelial cells	Esophagus, kidney, large intestine, liver, mammary gland, prostate, pituitary, salivary gland
	Mononuclear leukocytes	Most tissues examined
**Cytoplasmic localization with nuclear staining in some tissues**	Epithelial cells	Skin, tonsil, ureter, uterus
	Mononuclear leukocytes	Ovary
	Spindle cells	Adrenal gland, cervix, eye, heart, large intestine, liver, lymph node, lung, striated muscle, prostate, pituitary, salivary gland, skin, spleen, spinal cord, thymus, tonsil
	Glial cells and processes	Cerebellum, cerebral cortex, spinal cord
	Cell processes within retina	Eye
	Rare cells and processes	Peripheral nerve, heart, prostate
	Pituicytes	Pituitary neurohypophysis
	Vascular and intrinsic smooth myocytes	Cerebellum, cerebral cortex, cervix, esophagus, Fallopian tube, kidney, prostate, small intestine
	Skeletal myocytes	Striated muscle, prostate
	Vascular endothelium	Cervix, Fallopian tube, kidney, large intestine, liver, lymph node, lung, mammary gland, ovary, pancreas, prostate, salivary gland, small intestine, stomach, thyroid, thymus, uterus
	Lens fiber	Eye
	Adipocytes	Thyroid gland
**Extracellular localization**	Over stromal or interstitial elements or within the lumens of glands, kidney tubules, or vessels	Adrenal gland, bone marrow, cerebral cortex, kidney, large intestine, liver, lymph node, lung, mammary glands, striated muscle, peripheral nerve, placenta, pituitary, salivary gland, spinal cord, thymus, tonsil, ureter

Staining intensity and localization were assessed in tissue sections from human donors (*n* = 3 per tissue, where available.

scFv, single-chain variable fragment.

Similar cross-reactivity with tissues from NCG mice was observed (Table 2). Membrane staining often consisted of cytoplasmic lining the cell surface and was restricted to mononuclear leukocytes, and staining of extracellular material was observed in various tissues. Most of the observed staining was cytoplasmic in nature. Membrane staining was very rare or rare with staining observed in 5% or less of all mononuclear cells within the tissue.

**Table 2.** Cross-reactivity profile of SBT777101 scFv in normal NCG mouse tissues.

Cellular compartment	Cell type	Tissue
**Membrane and cytoplasmic localization, with nuclear staining in some tissues**	Mononuclear leukocytes	Liver, lymph node, peripheral nerve, prostate
**Cytoplasmic localization with nuclear staining in some tissues**	Hematopoietic cells	Bone marrow
	Epithelial cells	Pancreas (acini), prostate (periurethral gland), salivary gland (acini), skin (follicular and hair shaft), ureter (mucosa)
	Spindle cells	Cervix, joints (forelimb and hindlimb), heart, lymph node, mammary gland, skeletal muscle, peripheral nerve, placenta, prostate, pituitary, parathyroid, salivary gland, skin, thyroid, uterus
	Endothelial cells	Cervix, lung, pancreas, placenta, prostate, spinal cord, uterus
	Nerve fibers and processes	Spinal cord
	Chondrocytes	Hindlimb cartilage
**Extracellular localization**	Over stromal or interstitial elements or within the lumens of glands, kidney tubules, or vessels	Cerebral cortex, cervix, esophagus, eye, Fallopian tube, joints (forelimb and hindlimb), heart, kidney, liver, lung, peripheral nerve, pancreas, placenta, prostate, pituitary, skin, spinal cord, stomach, thyroid, ureter, uterus

Staining intensity and localization were assessed in tissue sections from NCG mice (*n* = 15).

NCG, NOD-*Prkdc^em26Cd52^Il2rg^em26Cd22^*/NjuCrl; scFv, single-chain variable fragment.

### *SBT777101 shows no* in vitro *reactivity to normal cells*

To confirm the maintenance of a noncytotoxic Treg phenotype and evaluate potential off-target reactivity, SBT777101 Tregs were screened against a diverse panel of human primary cells representative of various tissues and/or identified via tissue-cross reactivity studies and literature search as possibly expressing CitP. The cell line SNU449, which expresses high levels of the citrullinating enzyme PAD2 and vimentin and demonstrated some surface and intracellular binding of the SBT777101 CAR scFv by flow cytometry (Fig. 4A), was used as a control. UT Tregs and SBT777101 derived from 2 donors demonstrated no cytotoxicity against SNU449 cells (as measured by SNU449 viability), even in the presence of additional CAR stimulation with CV-coated beads, despite upregulation of the activation marker CD69 (Fig. 4B).

**Fig. 4.**
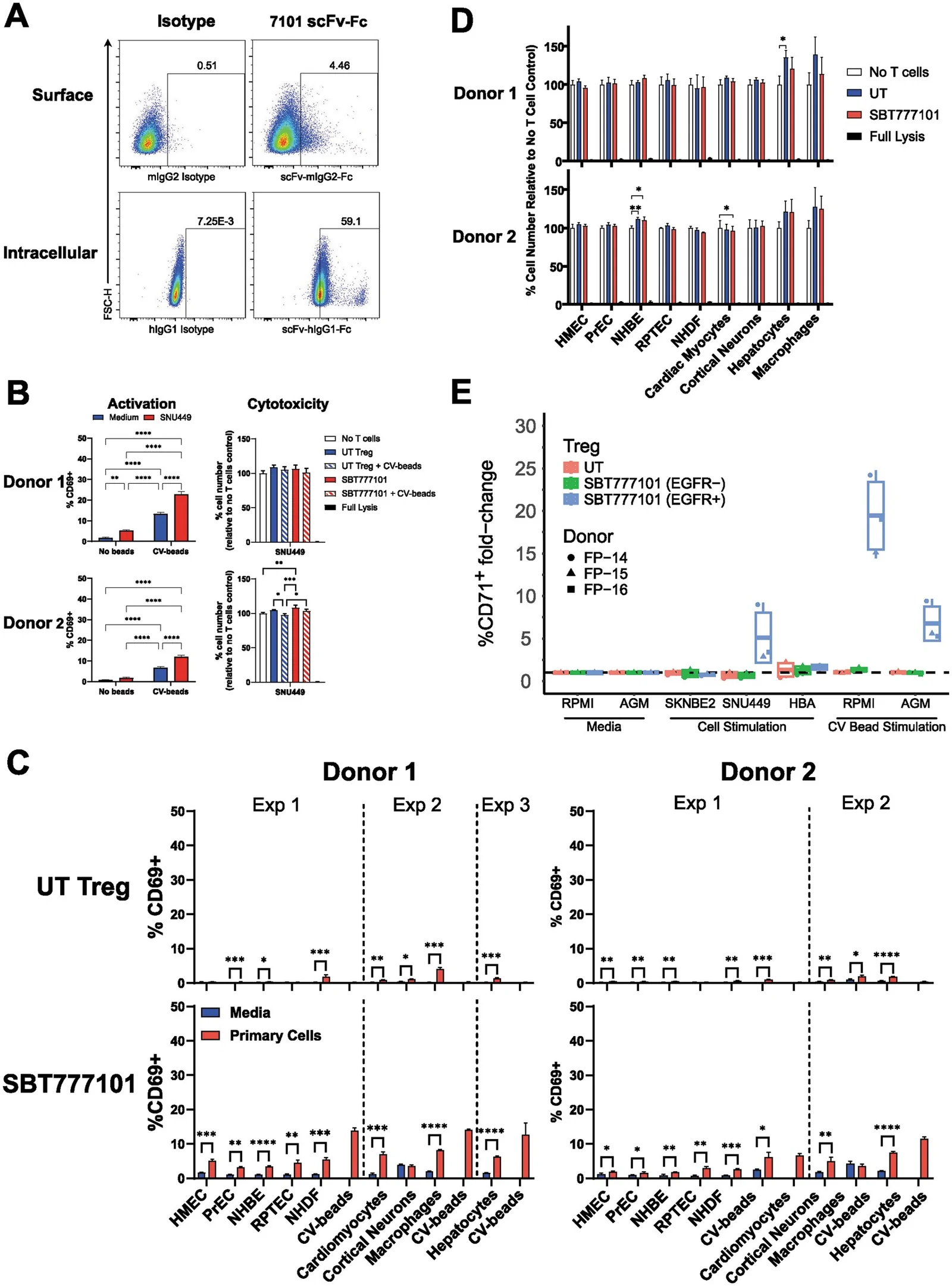
SBT777101 demonstrates minimal antigen-specific activation without impact on normal human cell survival. (A) Representative flow cytometry plots showing surface (top) and intracellular (bottom) binding of SBT777101 scFv-Fc (right) relative to isotype control (left) in SNU449 cells. (B) In vitro functional characterization using SNU449 cells ± CV-beads. Left: Mean ± SD CD69 expression (only SBT777101 data shown; no activation observed in UT controls). Data analyzed by two-way ANOVA with Sidak’s multiple comparisons test. Right: Cytotoxicity measured as the number of viable target cells remaining, normalized to no-Treg controls. Data analyzed by one-way ANOVA with Tukey’s multiple comparisons test. (C) Percentage of UT (top) or SBT777101 (bottom) Tregs expressing CD69 after 24-h co-culture with normal human primary cells or CV-beads (positive control). Data represent mean ± SD from three replicates. Independent experiments are separated by dashed vertical lines. Comparisons were made for media alone compared with in the presence of primary cells for each type and analyzed by two-tailed unpaired *t*-test. (D) Cytotoxicity (mean ± SD) of SBT777101 and UT Tregs against the same primary cell panel, normalized to no-Treg controls. Data analyzed by one-way ANOVA with Tukey’s multiple comparisons test. (E) Fold-change in CD71 expression (mean ± SD; relative to media control) across three donors after 72-h culture with media, CitP^−^ SKNBE2 cells, CitP^+^ SNU449 cells, human brain astrocytes (HBA), or CV-beads. CV beads were prepared in RPMI or AGM media as appropriate. Full lysis controls (B, D) represent target cells treated with Triton X-100. *P < 0.05; **P < 0.01; ***P < 0.001; ****P < 0.0001. All experiments included 3 technical replicates. AGM, astrocyte growth medium; CitP, citrullinated protein; CV, citrullinated vimentin; HMEC, human mammary epithelial cells; NHBE, normal human bronchial epithelial cells; NHDF, normal human dermal fibroblasts; PrEC, prostate epithelial cells; RPTEC, renal proximal tubule epithelial cells; scFv, single-chain variable fragment; UT, untransduced.

Surface and intracellular binding of the SBT777101 CAR scFv in primary human cells representing distinct organ systems (as detailed in Materials and Methods) was minimal (data not shown). Similar to the SNU449 cell line, some upregulation of CD69 on SBT777101 cells was observed in response to primary human cells (Fig. 4C); however, no cytotoxicity against this panel of cells was observed (Fig. 4D). Additionally, human brain astrocytes (HBA) were assessed due to their high intracellular expression of GFAP, which was shown to potently activate the SBT777101 CAR in its citrullinated form. Flow cytometry confirmed high intracellular expression of GFAP; however, surface and intracellular binding of the SBT777101 CAR scFv was minimal indicating a lack of CitP expression (data not shown). When UT or SBT777101 Tregs were co-cultured with HBA for 72 h, only minimal upregulation of the activation marker CD71 was observed, with an average of a 1.6-fold increase in CD71 expression in CAR^+^ cells compared with media alone, and lower than that observed with SNU449 cells or stimulation with CV-coated beads (Fig. 4E). The small increase in CD71 was not the result of CitP expression as it was observed in UT Treg and the untransduced fraction (EGFR^−^) of SBT777101.

### SBT777101 is well tolerated in NCG mice following single-dose administration

A single-dose toxicity study was conducted in normal NCG mice to assess the safety and tolerability of SBT777101. A total of 80 NCG mice (40 males and 40 females) were randomized to 4 groups (*n* = 10/sex/group) consisting of two sets per group scheduled for termination on Day 7 or Day 14 (*n* = 5/sex/group/timepoint). Groups 1 and 2 were administered intravenous (IV) cryopreservation media diluted in saline. Group 3 received 5.0 × 10^6^ CAR^−^/EGFR^+^ Treg cells per animal, and Group 4 received 5.0 × 10^6^ cells of SBT777101. Mice in groups 2 through 4 received an intraperitoneal dose of 160,000 U of human IL-2 on weekdays (Study Days 2, 3, 6–10, 13) to promote survival of SBT777101 or CAR^−^/EGFR^+^ Treg cells. Regular assessments for mortality, moribundity, body weights, and clinical observations were performed. All animals survived until their scheduled necropsy. There were no SBT777101- or CAR^−^/EGFR^+^ Treg-related effects on clinical observations, body weight, organ weights, gross necropsy observations, or histopathology (Day 14 histopathology summarized in Table S2). In samples from liver, lung, and spleen, SBT777101 vector-derived sequence levels as assessed by ddPCR were below the lower limit of quantification, with only some animals in Groups 3 and 4 showing detectable vector on Day 7 or 14, suggesting a lack of significant expansion and/or persistence under these conditions. SBT777101 sequences were not detected in brain and blood on either Day 7 or 14 (Fig. S1). Human T cells were below the limit of detection in the blood by flow cytometry at both necropsy timepoints (data not shown).

### Lineage stability of SBT777101 is preserved following repeated stimulation including under proinflammatory conditions

The stability of the SBT777101 Treg phenotype was assessed in vitro by serially stimulating SBT777101 Tregs through the CAR or TCR weekly for 4 wk in the presence or absence of proinflammatory cytokines that mimic the synovial fluid in RA patients (Fig. 5A). Following each round of activation, SBT777101 FOXP3 expression remained high, regardless of the route of stimulation or presence of proinflammatory cytokines, with the average FOXP3^+^ percentage greater than 90% across all conditions, and greater than 80% for all individual donors on Day 28 (Fig. 5B).

**Fig. 5.**
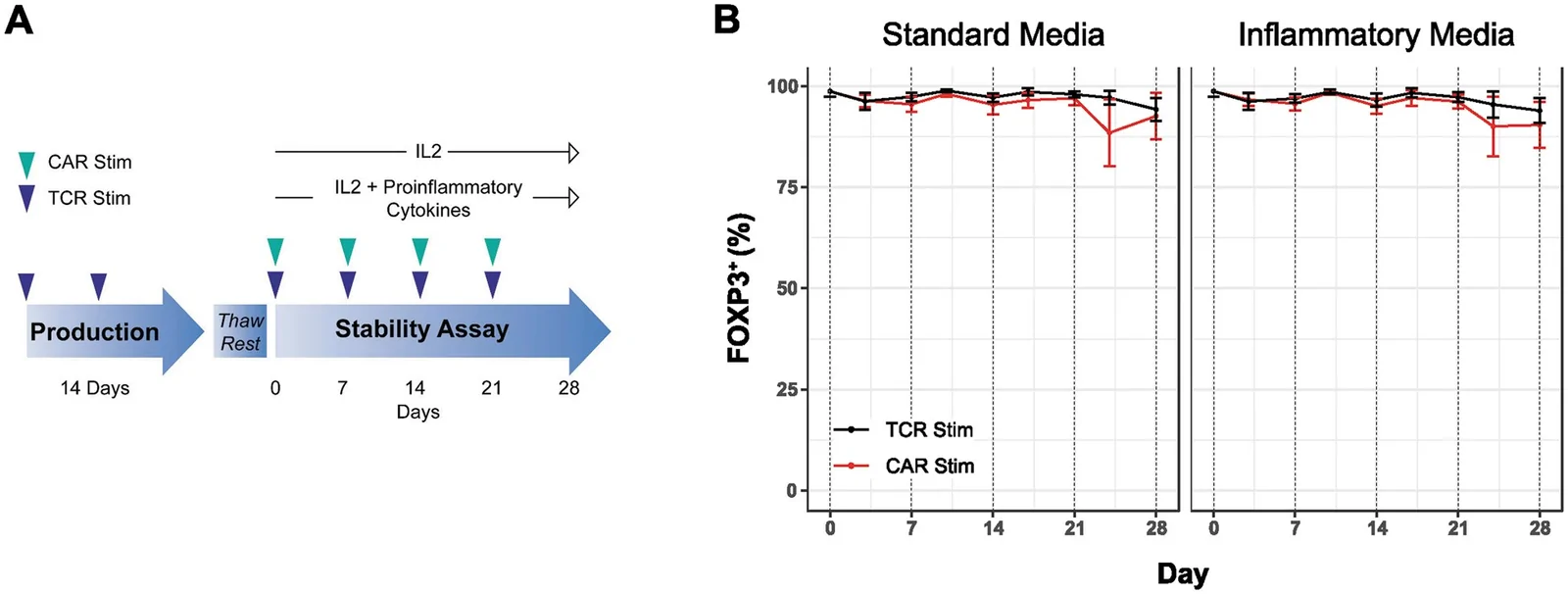
SBT777101 maintains phenotypic stability and lineage commitment under chronic inflammatory challenge. (A) Schematic of the 28-d stability assay. SBT777101, UT Treg, and UT Teff were subjected to four weekly stimulations via the endogenous TCR (anti-CD3/CD28) or the CAR (CV-beads) in the presence or absence of a proinflammatory cytokine cocktail. (B) Mean ± SD longitudinal FOXP3 expression (%) in SBT777101.

In vivo stability of the SBT777101 Treg phenotype was assessed in a GvHD model in NCG mice. In this study, irradiated mice were administered human PBMC to initiate GvHD. A subset of groups also received intranasal lipopolysaccharide (LPS) to promote local pulmonary inflammation; a prior experiment demonstrated a modest increase in SBT777101 CAR scFv staining in lung tissue following LPS administration in this model (Table S3). CAR^−^EGFR^+^ or SBT777101 Tregs were administered IV 24 h after human PBMC, and analysis was conducted on Day 13 (cytokine analysis) or Day 14 (histology analysis) (Fig. 6A). The administration of SBT777101 was associated with a marked reduction of GvHD-related proinflammatory human cytokines such as IFN-γ, which was similar to the reduction caused by CAR^−^EGFR^+^ Tregs (Fig. 6B). While no GvHD clinical scores were recorded in any group throughout the study, there was microscopic evidence of GvHD (perivascular infiltrate) in the spleen and/or lungs in groups given human PBMCs. In mice given CAR^−^EGFR^+^ or SBT777101 Tregs, there was a slight reduction in the incidence/severity of these findings compared with mice that received PBMCs alone (Table 3). Importantly, LPS administration and GvHD did not significantly affect the SBT777101 Treg phenotype—greater than approximately 70% of all EGFR^+^ cells recovered from the spleen at the end of the study were FOXP3^+^ (Fig. 6C).

**Fig. 6.**
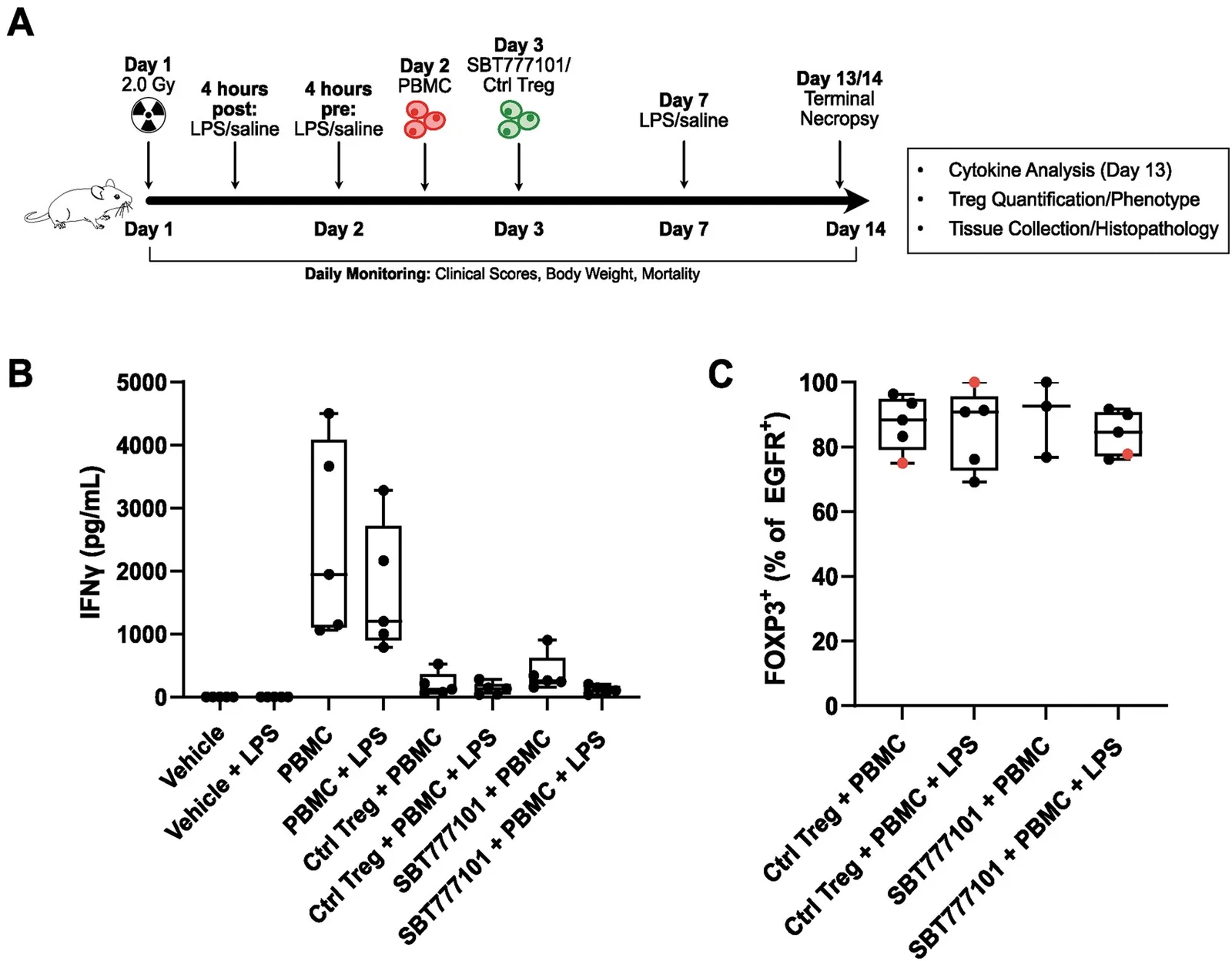
SBT777101 suppresses systemic inflammation and demonstrates lineage stability in a xenogeneic GvHD model. (A) Schematic of the xenogeneic GvHD model and treatment timeline. Sublethally irradiated mice (N = 5/group) received repeated intranasal LPS (or saline) challenges (Days, 1, 2, and 7) to simulate an inflammatory environment. Disease was initiated via IV infusion of human PBMCs (or vehicle control) on Day 2. Therapeutic cells (SBT777101 or Ctrl [CAR^−^EGFR^+^] Treg) were administered on Day 3, with terminal necropsy on Day 13 or 14. (B) Serum was collected on Day 13 to evaluate the systemic inflammatory response. Cytokine levels (pg/ml) are presented as box and whisker plots showing the median and interquartile range; whiskers represent minimum and maximum values. (C) Splenocytes were harvested at necropsy (Day 14) to evaluate the phenotypic stability of the infused cells. Data represent the percentage of FOXP3^+^ cells within the transduced (EGFR^+^) population. Points highlighted in red denote samples with fewer than 10 EGFR^+^ events.

**Table 3.** Summary of GvHD-related histopathological findings in NCG mice following human PBMC transfer and LPS challenge.

Tissue	Finding	Grade	Group (*n* = 5/group)							
			Vehicle	Vehicle + PBS	PBMC	PBMC+LPS	Ctrl + PBMC	Ctrl + PBMC+LPS	7101 + PBMC	7101 + PBMC+LPS
**Spleen**	Infiltrate, mononuclear, perivascular	Any	–	–	5	5	4	4	2	4
		1	–	–	2	2	2	4	1	2
		2	–	–	2	3	2	–	1	2
		3	–	–	1	–	–	–	–	–
**Lung**	Infiltrate, mixed cell, perivascular	1	–	–	–	–	–	1	1	–

7101 = SBT777101; Ctrl, CAR^−^EGFR^+^ Treg; LPS, lipopolysaccharide; PBMC, peripheral blood mononuclear cells; PBS, phosphate-buffered saline.

### SBT777101 exhibits a regulatory T cell cytokine profile supporting a low risk of cytokine release syndrome

Treatment with CAR-Teff cells in the clinic is associated with a significant risk of cytokine release syndrome (CRS) resulting from potent activation and proinflammatory cytokine secretion (Frey and Porter 2019). While Tregs are known to have a cytokine secretion profile distinct from Teff (Levings et al. 2001), secreting small amounts of proinflammatory cytokines and larger amounts of anti-inflammatory cytokines such as IL-10, we assessed whether the manufacturing process altered this profile.

Expanded UT Teff and SBT777101 Tregs were generated from healthy donors and from patients with RA or HS, then stimulated through the TCR or the CAR (SBT777101 only). Upon stimulation through the TCR, expanded Teffs produced high levels of GM-CSF, IFN-γ, IL-10, IL-17A, and TNF-α. SBT777101 Tregs secreted, on average, lower amounts of each of the five cytokines compared with Teff; among them, IL-10 was secreted at the highest concentration. Importantly, proinflammatory cytokine secretion from SBT777101 Tregs was consistent regardless of stimulation method (CAR vs TCR) or donor disease status, and remained substantially lower than that of Teff cells, suggesting a decreased risk of CRS compared with CAR Teff cell therapies (Fig. 7).

**Fig. 7.**
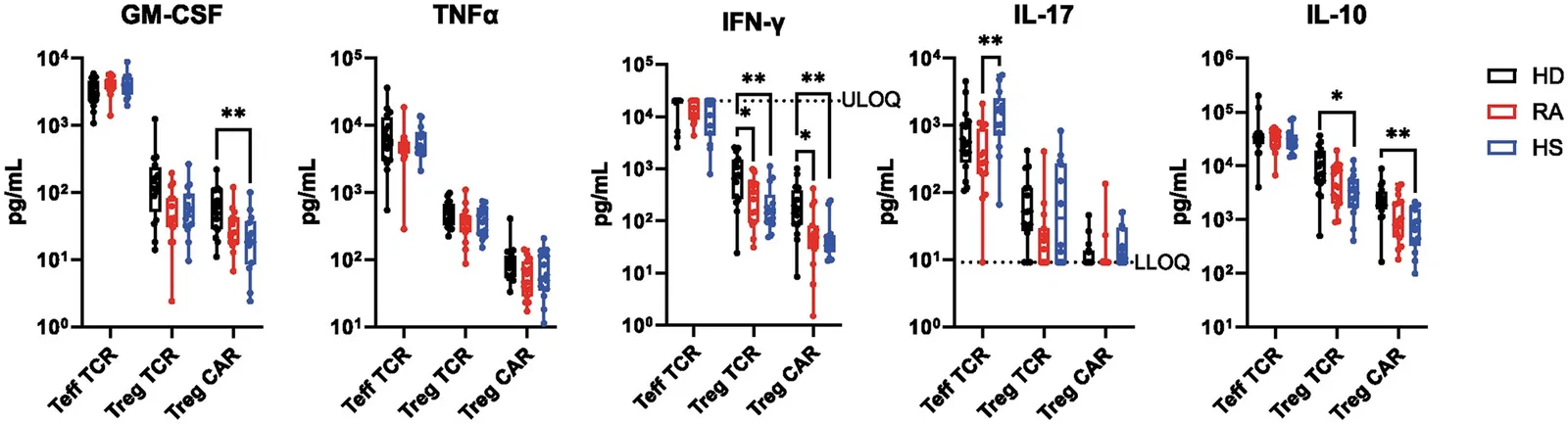
Cytokine profile of SBT777101 derived from healthy donors and patients with autoimmune disease. Effector T cells (Teff) and SBT777101 were derived from healthy donors or patients with rheumatoid arthritis (RA) or hidradenitis suppurativa (HS). Cytokine concentration (pg/ml) for GM-CSF, TNF-α, IFN-γ, IL-17, and IL-10 were measured following stimulation via the endogenous TCR (anti-CD3/CD28) or the CAR (CV-beads). Statistical significance was determined by Kruskal–Wallis test followed by Dunn’s multiple comparisons *post hoc* test. *P < 0.05; **P < 0.01.

### SBT777101 preserves innate NK cell function and requires preactivation for partial virus-specific CD8^±^ T cell inhibition

While polyclonal Treg cell therapies have not shown signs of systemic immunosuppression to date (Duggleby et al. 2018), these studies sought to confirm that the addition of antigen-specificity does not increase the risk of compromising appropriate immune responses to malignancy or infection.

To assess the effect of SBT777101 Tregs on host antitumor surveillance, their impact on NK cell-mediated cytotoxicity was evaluated using the well-established K562 erythroleukemic target cell model. The suppressive potential of UT or SBT777101 Tregs was assessed under three distinct physiological scenarios toward autologous NK cells. The potential for inherent suppression in the absence of antigen was assessed using resting cells. To simulate a scenario where a CAR-Treg has been fully primed by its cognate antigen before encountering a target, the impact of preactivated Tregs (stimulated for 72 h via CAR [SBT777101 only] or TCR) was examined. Finally, to evaluate the effect of immediate, in situ engagement in an antigen-rich environment, concomitant activation was tested by stimulating resting Tregs through the CAR or TCR at the time of co-culture with NK and K562 cells. Under each of these conditions, SBT777101 did not suppress NK cytotoxicity against K562 cell lines (Fig. 8).

**Fig. 8.**
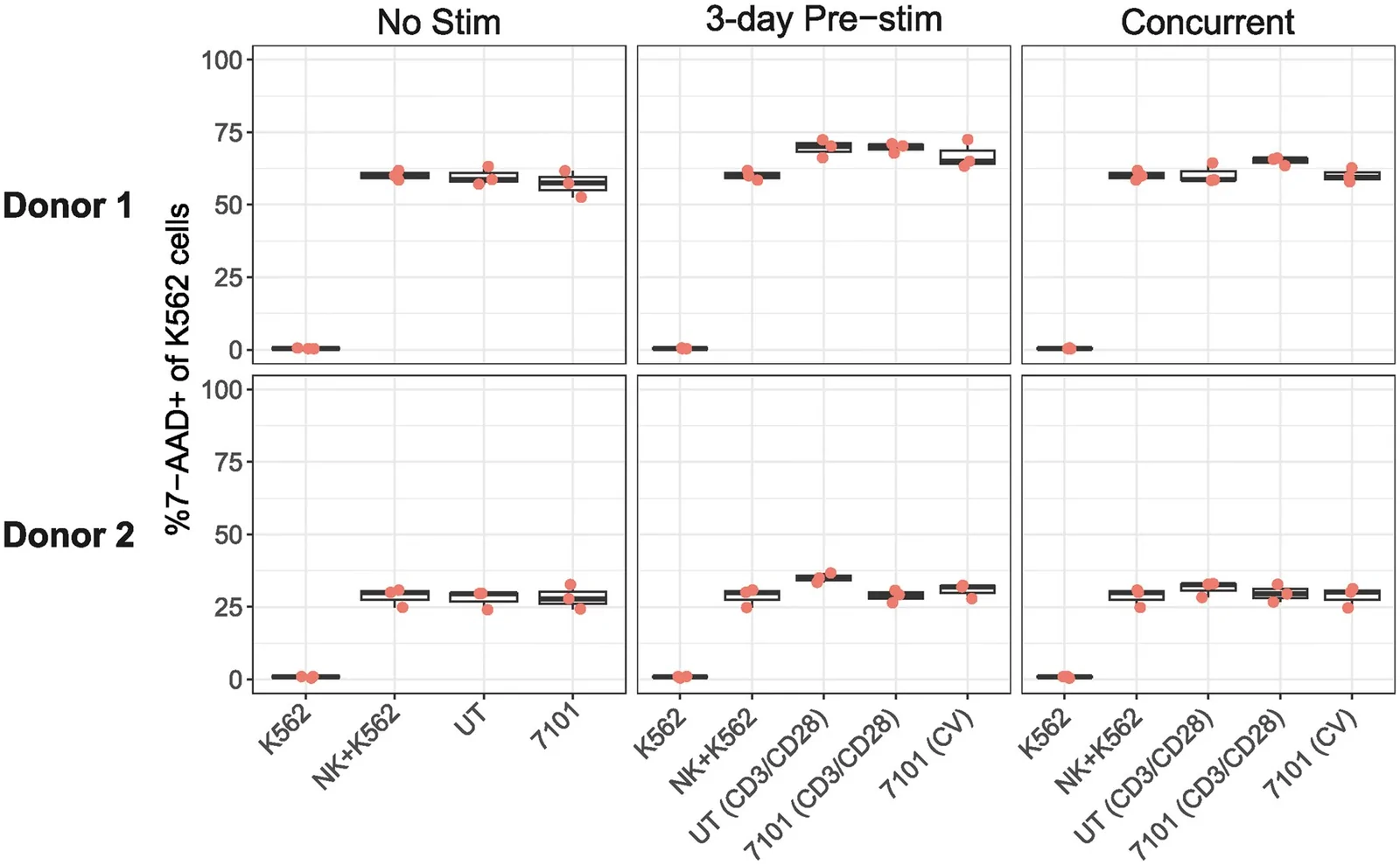
Impact of SBT777101 on NK cell-mediated cytotoxicity. Percentage of nonviable K562 target cells following 24-h co-culture with NK cells and either UT or SBT777101 (7101) Tregs (1:1 Treg: NK ratio). Viability was determined by 7-AAD staining and flow cytometry. Experimental conditions include: (Left) No exogenous stimulation; (Middle) Tregs prestimulated for 24 h with anti-CD3/CD28 (TCR) or CV-beads (CAR; SBT777101 only) prior to co-culture; and (Right) concurrent TCR or CAR stimulation during the co-culture assay. Each data point represents an individual replicate across two independent donors.

Similarly, the potential for SBT777101 to interfere with protective T cell immunity was evaluated by assessing its impact on the recall response of autologous CD8^+^ effector cells. Autologous CD8^+^ T cells and monocyte-derived DCs were co-cultured with viral-derived recall antigens in the presence of UT or SBT777101 Tregs under the same functional conditions as the NK suppression study. Following 48 h of incubation, IFN-γ secretion was quantified in the supernatants. While resting and concomitantly activated Tregs did not inhibit the antiviral response, SBT777101 Tregs that were preactivated for 72 h through either the TCR or CAR partially inhibited CD8^+^ T cell IFN-γ production. Comparable decreases were observed with TCR preactivated UT Tregs, indicating that this partial suppression is a characteristic of the underlying polyclonal Treg biology rather than a consequence of CAR engineering and requires full activation of the cells prior to encountering CD8^+^ T cells (Fig. 9). Collectively, these data suggest that SBT777101 maintains a safety profile consistent with polyclonal Tregs.

**Fig. 9.**
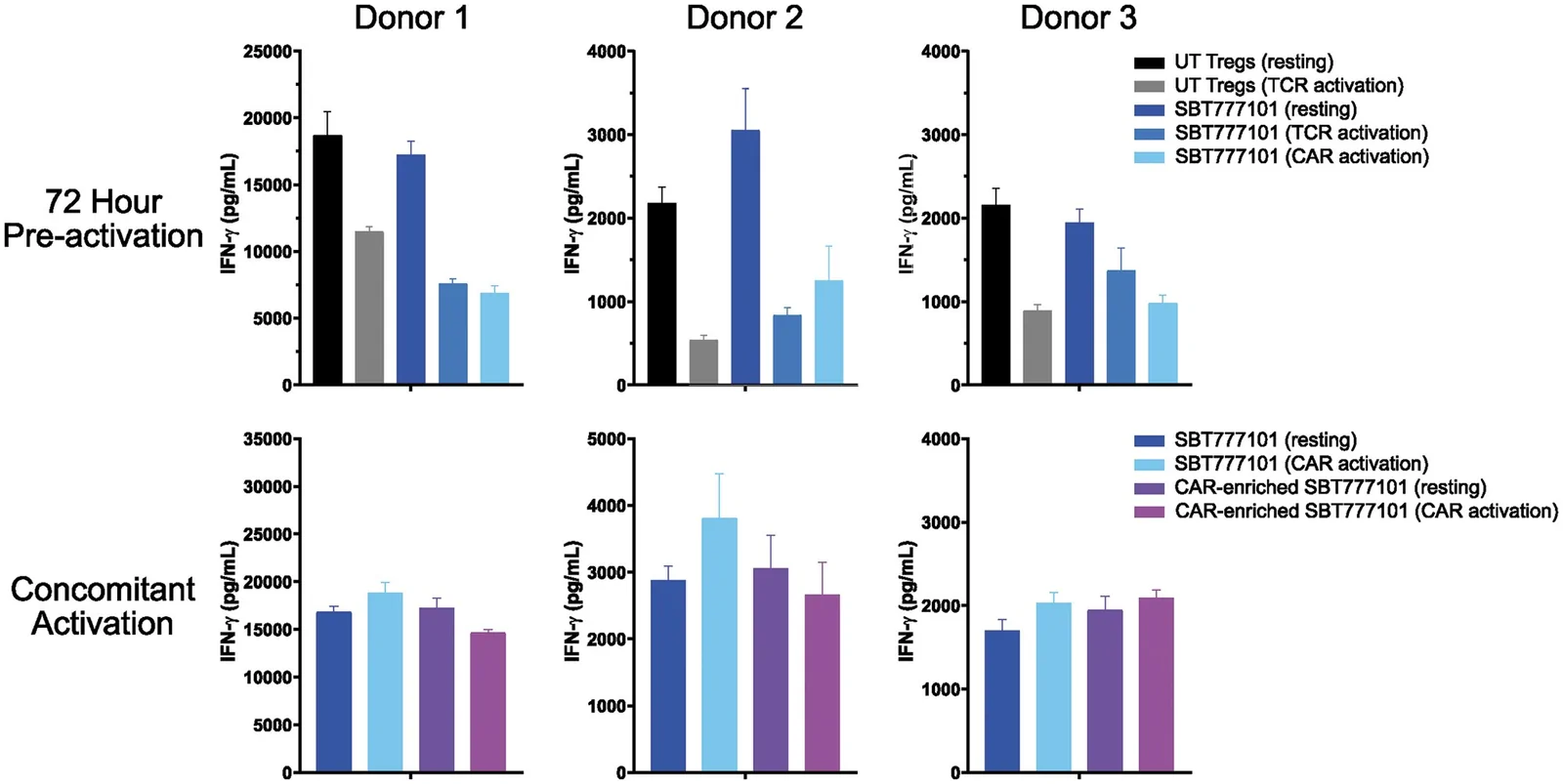
IFN-γ production by antigen-specific CD8^+^ T cells in co-culture with SBT777101. IFN-γ concentrations were measured in cell culture supernatants following 48 h of culture of untransduced (UT) or SBT777101 Tregs with CD8^+^ T cells and monocyte-derived dendritic cells in the presence of an MHC-I-restricted peptide mix (CERI). (Top) Tregs were either rested or prestimulated with anti-CD3/CD28 beads or CV-beads (SBT777101 only) for 72 h prior to co-culture. (Bottom) SBT777101 or CAR-enriched SBT777101 were stimulated concurrently with CV-beads during co-culture. All data represent the mean ± SD of three technical replicates.

### Citrullinated proteins recognized by the SBT777101 scFv are minimally expressed in malignant and infectious tissues and comparable to healthy controls

To determine the potential for on-target/off-disease activation in the context of malignancy or infection, the binding profile of the SBT777101 scFv was evaluated across a panel of malignant and infectious disease-derived tissues where citrullination may be pathologically induced (Table 4). In malignant solid and lymphoid tumor samples, the observed staining was primarily cytoplasmic and nuclear, and mostly consistent with patterns seen in corresponding normal tissues. An increased frequency of nuclear and/or cytoplasmic staining was noted in mononuclear leukocytes in cerebral, ovary, kidney, lung, and prostate tumors relative to normal samples; this was consistent with a higher density of inflammatory cells identified by hematoxylin and eosin (H&E) staining in these tissues. While extracellular staining was absent in normal lymph nodes, it was observed in 3 of 9 B-cell lymphoma samples.

**Table 4.** Tissue binding profile of SBT777101 in selected malignant and infectious disease-derived tissues.

Category	Tissue/condition	Localization	Cell types with staining	Compared to normal	Notes
Tumor	Cerebrum	Cytoplasmic & nuclear	Mononuclear Leukocytes	Increased	Increased inflammatory cells noted by H&E
	Ovary	Cytoplasmic & nuclear	Mononuclear Leukocytes	Increased	Increased inflammatory cells noted by H&E
	Kidney	Cytoplasmic & nuclear	Mononuclear Leukocytes	Increased	Increased inflammatory cells noted by H&E
	Lung	Cytoplasmic & nuclear	Mononuclear Leukocytes	Increased	Increased inflammatory cells noted by H&E
	Prostate	Cytoplasmic & nuclear	Mononuclear Leukocytes	Increased	Increased inflammatory cells noted by H&E
	B-cell lymphoma	Extracellular	Extracellular material	Increased	Extracellular staining in 3/9 samples; not seen in normal lymph node
Infection	HBV liver	Cytoplasmic & nuclear	Mononuclear leukocytes, epithelial	Comparable	Staining similar to normal liver tissue
	HPV cervix	Cytoplasmic & nuclear	Mononuclear leukocytes, epithelial	Increased	Increased inflammatory cells noted by H&E
		Extracellular	Extracellular material	Increased	Extracellular staining in 1/3 samples; not seen in normal cervix
	TB lung	Cytoplasmic & nuclear	Mononuclear leukocytes	Comparable	Staining similar to normal lung tissue
		Cytoplasmic & nuclear	Epithelial	Increased	Staining in 3/3 samples; not seen in normal lung
		Extracellular	Extracellular material	Increased	Staining in 3/3 samples; not seen in normal lung

Staining intensity and localization were assessed in human tissue microarrays (*n* = 5 each) consisting of normal tissues, normal and malignant lymphocytes, cancer tissues, and normal and HBV-infected liver, as well as *n* = 3 HPV cervix and TB lung tissues. H&E, hematoxylin and eosin; HBV, hepatitis B; HPV, human papillomavirus; TB, tuberculosis.

Similarly, SBT777101 scFv binding in chronic infection tissues was primarily nuclear and cytoplasmic within epithelial and inflammatory cells across all three indications evaluated (hepatitis B virus [HBV] liver, human papillomavirus [HPV] cervix, and tuberculosis [TB] lung). Staining in HBV-infected liver was comparable to normal liver controls. However, HPV-infected cervical tissue showed increased epithelial and inflammatory cell staining compared with normal cervix, with extracellular staining present in one of three samples. In TB-infected lung, staining was identified in epithelial cells, inflammatory cells, and extracellular material across all three samples, whereas in normal lung, staining was limited to mononuclear leukocytes. Overall, these findings indicate that while some pathological citrullination occurs in malignancy and chronic infection, the resulting antigenic burden is primarily intracellular or localized.

### SBT777101 demonstrates a typical Lentivirus-associated insertion profile and lack of cytokine-independent proliferation

As a critical safety requirement for genetically modified cell therapies, the potential for tumorigenicity and insertional mutagenesis was evaluated in the final SBT777101 product. These studies focused on characterizing the lentiviral integration profile and demonstrating a lack of abnormal outgrowth in the absence of activation and cytokines.

Integration site (IS) analysis was used to assess the clonality of lentiviral vector integration within the SBT777101 product from 4 individual donors. The analysis yielded high sequencing depth, with a total of 73,105 unique, exactly mappable IS identified across the samples. All samples demonstrated a highly polyclonal integration profile, as evidenced by a polyclonal–monoclonal distance (PMD) index consistent with diversity in vector integration sites. Importantly, no evidence of clonal dominance was observed; the relative contribution of any single integration site remained below 0.4%, well under the 30% threshold typically used to define dominant clones. While 16.07% of the identified IS were located near genes listed in the Cancer Gene Census database, the overall occurrence of these events was low and distributed across the population. Furthermore, though 95.4% of IS were found within common insertion sites clustered within 50 kbp, the absence of skewed retrieval frequencies suggests a lack of selective expansion. Collectively, these data confirm that the SBT777101 vector integrates polyclonally without the emergence of dominant, potentially transformed clones (Fig. 10).

**Fig. 10.**
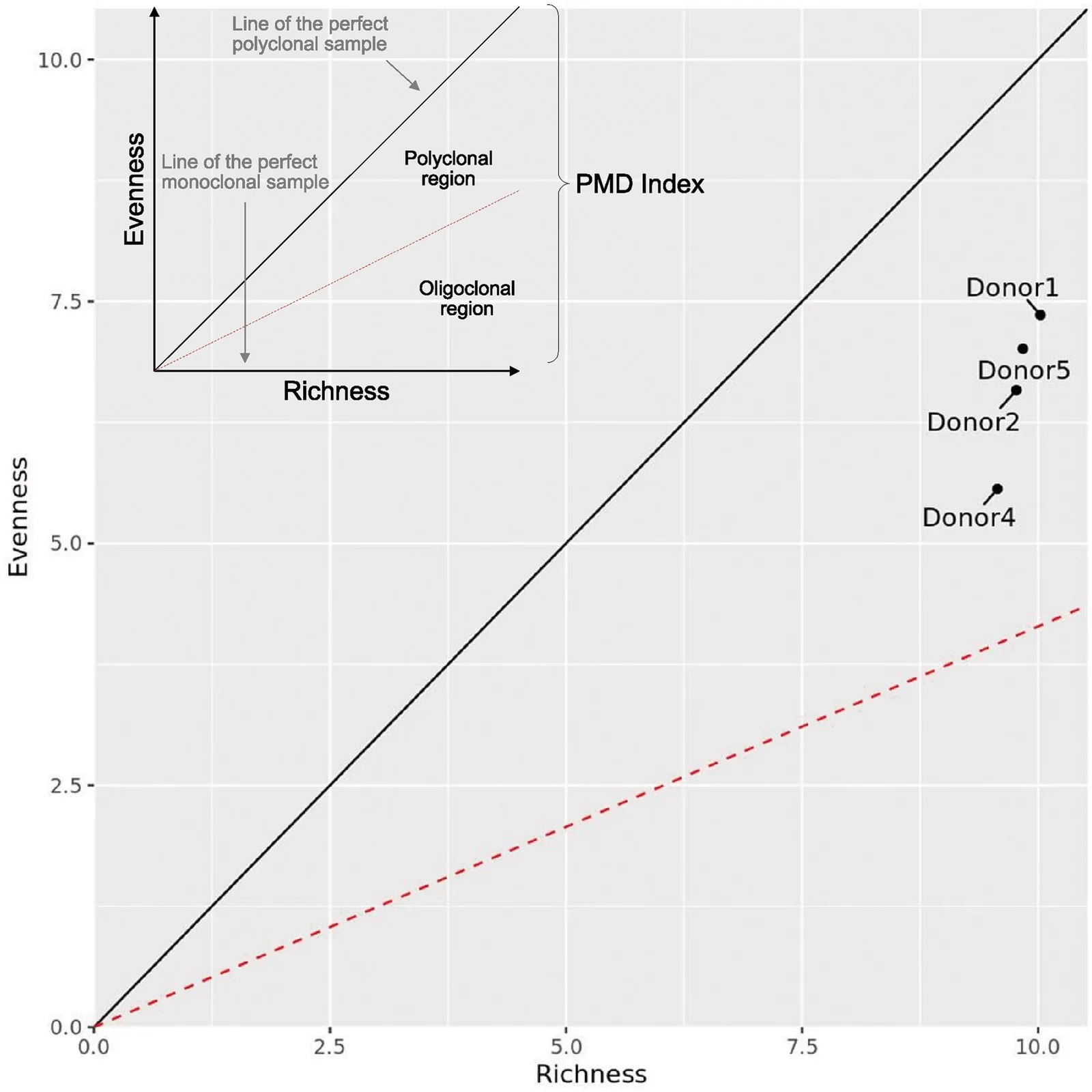
Polyclonal–monoclonal distance (PMD) analysis of integration site diversity. PMD values are based on Rényi entropy-derived measures of richness and evenness, plotted in a two-dimensional clonality plane. The PMD value reflects the relative distance of each sample from the theoretical maximum of polyclonality (inset schematic, top left). Data from 4 representative donors are shown.

**Fig. 11.**
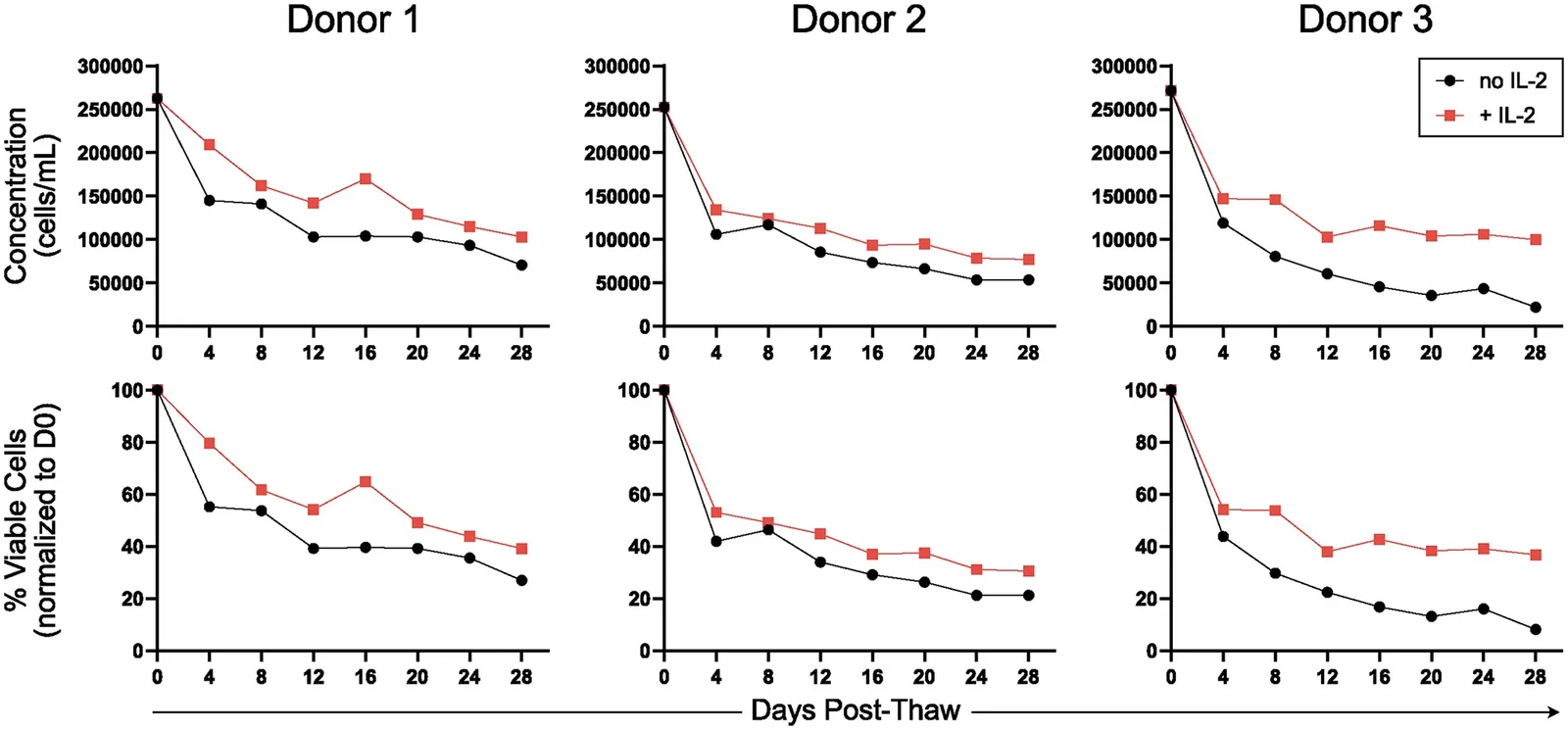
Influence of IL-2 on the survival and proliferation of SBT777101. Cell concentration (top) and viability (bottom) of SBT777101 Tregs derived from three donors during a 28-d culture period in the absence of exogenous TCR or CAR stimulation. Cells that were supplemented with IL-2 received cytokine every 4 d. Cell concentration is presented as cells/ml. Viability was determined by AO/DAPI and is shown as a percentage of viable cells normalized to Day 0 values.

To complement the genomic IS analysis, the functional growth requirements of SBT777101 were evaluated to ensure the maintenance of homeostatic control and signal-dependent proliferation. SBT777101 cells from 3 donors were cultured without TCR or CAR stimulation in the presence or absence of exogenous IL-2 for 28 d. No proliferation was observed in any sample; while the addition of IL-2 improved survival compared with cytokine-free controls, overall cell numbers and viability steadily decreased over the 28-d period (Fig. 11). This lack of autonomous expansion, combined with the diverse integration profile, demonstrates that the SBT777101 manufacturing process does not induce malignant transformation or cytokine-independent growth.

## Discussion

CAR-Treg cell therapies represent an emerging class of investigational products for which nonclinical safety frameworks are still evolving, as engineering antigen specificity into Tregs introduces complexities poorly captured by conventional model systems. CAR-Treg pharmacology can be antigen-, disease-, and species-specific, limiting the utility of standard in vitro and in vivo toxicology models. The nonclinical program for SBT777101, a CAR-Treg targeting CitP to suppress inflammation in RA and HS, was built around in vitro and ex vivo NAMs, benchmarked against UT polyclonal Tregs, which have been well toleratedacross more than 40 completed human studies (Bluestone and Tang 2018). Following Health Authorities interactions, these NAMs were complemented with in vivo studies in immunodeficient animals to assess effects on tissues expressing CitP, off-disease activation risk, and phenotype stability under active inflammation.

A central safety question is whether CAR engineering and a proinflammatory environment can destabilize the Treg phenotype, converting a suppressive cell into an inflammatory effector (Bailey-Bucktrout et al. 2013). FOXP3 stability, a defining feature of the Treg phenotype, was confirmed in vitro following repeated stimulations with proinflammatory cytokines elevated in RA, and in vivo in the presence of human proinflammatory cytokines produced during GvHD, demonstrating stability similar to that of UT Tregs. This indicates a low likelihood that the SBT777101 Treg phenotype would be altered in the RA context or exacerbate disease—consistent with clinical observations that similarly expanded polyclonal Tregs maintained phenotype over time in T1D (Bluestone et al. 2015). Upon CAR or TCR stimulation, SBT777101 produced low levels of proinflammatory cytokines in contrast to Teff cells, a finding consistent across RA-, HS-, and healthy donor-derived products, suggesting a low risk of CRS. Treg-specific demethylated region (TSDR) analysis, an orthogonal marker of stable Treg lineage commitment, was used for product quality characterization but not incorporated into this evaluation; FOXP3 expression across repeated stimulation was considered sufficient evidence of phenotypic stability, though TSDR analysis could be considered in future work.

Taken together, these data suggest a safety profile similar to that of polyclonal expanded Tregs, with no indication of exacerbated inflammation upon CAR engagement. Theoretical risk of CRS was considered, though it has not been reported with autologous or allogeneic polyclonal Tregs across autoimmune or transplant indications (Raffin et al. 2020). In CAR-Teff therapy, circulating CAR^+^ Tregs at peak cell expansion have been associated with milder, CRS-linked neurotoxicity, suggesting a potentially neuroprotective role for CAR-Tregs (Good et al. 2022).

A key safety liability with antigen-specific CAR-Treg therapies is on-target off-disease activity in tissues expressing the target antigen outside the disease context (Morgan et al. 2010). Citrullination, conversion of arginine to citrulline by peptidylarginine deiminase (PAD) enzymes, is markedly elevated in RA (Darrah and Andrade 2018) and HS (Byrd et al. 2019) but occurs at low levels in normal tissues (Baka et al. 2012). Tissue cross-reactivity studies using an SBT777101 scFv fusion protein revealed that most binding was cytoplasmic or nuclear, which is of little toxicologic relevance given the limited intracellular access of cell-based therapeutics (Hall et al. 2010; Leach et al. 2010). Membrane binding was rare, limited to epithelial cells in select tissues and mononuclear leukocytes, and often reflected cytoplasmic granules lining the cell surface rather than a distinct membrane border, suggesting minimal concern. Extracellular binding, observed across many tissues, was frequently associated with stromal or interstitial elements that could not be co-localized to a specific cell type, and was also present in glandular secretions, kidney tubule lumens, and vessels, with unknown toxicological relevance (Hall et al. 2010; Leach et al. 2010). This analysis may not capture inducible citrullination arising in inflamed nontarget tissues under unrelated inflammatory conditions, a residual uncertainty relevant to clinical monitoring. The scFv binding profile was also evaluated across malignant and infectious disease-derived tissues where citrullination may be pathologically induced, indicating that antigenic burden in malignancy and chronic infection is primarily intracellular or localized, though inducible citrullination outside the target disease remains part of ongoing clinical safety surveillance.

To directly evaluate functional activation in normal cell contexts, the activation and cytotoxicity of SBT777101 were assessed against normal human primary cells and the SNU449 cell line, which expresses high PAD2 and vimentin and served as a control. SBT777101 showed no cytotoxicity toward any cells tested, consistent with Treg stability and absence of effector function (Overacre and Vignali 2016). Because SBT777101 can be activated by Cit-GFAP, and GFAP is expressed in astrocytes (Hol and Pekny 2015), primary human astrocytes were evaluated and showed minimal scFv binding and no CAR-specific activation. In vivo, no SBT777101 activation was observed in NCG mice, where scFv binding to mouse tissues resembled human tissues. These findings support a low likelihood of on-target off-disease activity.

The possibility of off-target effects was similarly low: The SBT777101 scFv showed no off-target binding to 5,828 individual full-length human plasma membrane proteins and cell surface-tethered secreted proteins, nor to 398 human heterodimeric protein complexes, in a comprehensive protein array study. A limitation of this array is that it lacks posttranslationally citrullinated proteins.

The potential for unwanted immunosuppression—difficult to model in standard animal systems given species-specific differences in immune regulation—was assessed by comparing SBT777101 immunomodulatory activity to UT polyclonal Tregs. SBT777101 showed no impact on NK cell cytotoxicity against the K562 line, and inhibited antiviral CD8^+^ T cell responses only when fully activated prior to encountering CD8^+^ T cells, mirroring the behavior of UT polyclonal Tregs . scFv binding in malignant and infectious disease-derived tissues did not show an appreciable increase in citrullinated proteins in disease contexts requiring a competent immune response. In vivo, low target antigen expression in normal tissues did not result in activation, and GvHD model immunomodulatory activity was comparable to UT polyclonal Tregs. Collectively, these data indicate that SBT777101 did not exhibit greater nonspecific immunosuppressive activity than UT polyclonal Tregs under the conditions evaluated. Because CAR-Tregs differ from polyclonal Tregs in antigen specificity, activation biology, and persistence, definitive conclusions regarding clinical immunosuppression risk await clinical data. This nonclinical finding is further supported by the favorable clinical safety record of polyclonal Treg therapies across autoimmune and transplant indications, including T1D (Marek-Trzonkowska et al. 2012; Bluestone et al. 2015), kidney (Mathew et al. 2018; Sawitzki et al. 2020) and liver (Todo et al. 2016; Sanchez-Fueyo et al. 2020) transplantation, Crohn’s disease (Desreumaux et al. 2012), systemic lupus erythematosus (Dall’Era et al. 2019), pemphigus (Plaisse et al. 2026), and graft versus host disease (Trzonkowski et al. 2009; Meyer et al. 2025), where few SAEs were observed. Those that were reported were consistent with the underlying disease, and most events resolved without sequelae.

Concerns regarding lentiviral vector-modified cell products include the potential for insertional mutagenesis caused by integration of the proviral DNA and viral promoters near active genes (Shah et al. 2019). All transduced samples exhibited polyclonal integration profiles with no dominant sites. SBT777101 numbers and viability declined over 28 d in culture with or without exogenous IL-2, with no abnormal growth observed absent CAR or TCR stimulation. No evidence of clonal dominance, abnormal proliferative behavior, or malignant transformation was observed under the conditions examined. As with other lentivirally modified therapies, a residual theoretical risk of insertional mutagenesis cannot be fully excluded by preclinical studies alone, underscoring the importance of clinical surveillance.

This assessment lacked an animal model with a fully functional immune system capable of demonstrating antigen-specific SBT777101 activation. The antigen- and species-specificity of CAR-Treg products, and the inability of standard rodent models to recapitulate the inflammatory milieu of human RA or HS, necessitated the NAM-based approach described here (Shenton et al. 2025). While these data do not indicate an increased safety risk from engineering antigen specificity into SBT777101, the translational validity of these in vitro and ex vivo approaches remains to be established. Correlating nonclinical findings with clinical safety observations in Phase 1 studies will be critical for determining whether this testing paradigm is fit for purpose and can serve as a framework for future CAR-Treg development programs.

Unlike conventional nonclinical programs for nononcology indications, where the FIH dose is typically derived from a no-observed-adverse-effect-level (NOAEL), SBT777101 dose selection followed a different approach given the limited translational relevance of standard toxicology models for CAR-Treg pharmacology. The in vivo tolerability and GvHD studies were designed to include dose levels exceeding the proposed clinical dose on a cells/kg basis, but because CAR-Treg activity is antigen-, disease-, and species-specific, a NOAEL-based margin was not used to derive the FIH dose directly. Instead, the FIH dose was selected empirically from extensive clinical experience with UT polyclonal Tregs, informed by the totality of the nonclinical package demonstrating that SBT777101 does not exhibit a safety profile distinct from UT Tregs. This approach—establishing safety comparability to a clinically established cell therapy across in vitro and in vivo NAMs, rather than a NOAEL-derived margin—provided the basis for dose selection.

In conclusion, the nonclinical safety assessment of SBT777101 supported initiation of Phase 1 studies in RA (NCT06201416) and HS (NCT06361836). Critically, the totality of data demonstrates that engineering antigen specificity via a CAR does not appreciably alter the safety profile of SBT777101 relative to polyclonal Tregs, supporting the broader tractability of this approach for future antigen-specific Treg development.

## Supplementary Material

kfag125_Supplementary_Data

## Data Availability

Data are available via the corresponding author upon reasonable request.
