# Supplementary material for: Nonclinical safety assessment of SBT777101, a CAR-engineered regulatory T cell therapy for autoimmune diseases
Source: Toxicol Sci. 2026 Sep 15;209(10):kfag125. doi: 10.1093/toxsci/kfag125 (PMC13637847; doi:10.1093/toxsci/kfag125)
Supplement: Supplementary file 1 [file kfag125_supplementary_data.docx]

**Nonclinical Safety Assessment of SBT777101, a CAR-Engineered Regulatory T Cell Therapy for Autoimmune Diseases**

Herve Lebrec^1^, Jonathan M. Clingan^1^, Anne-Renee van der Vuurst de Vries^1^, Lauren Mihalcik^2^, Yuchi Honaker^1^, Sara Charmsaz^1^, Seonhwa Dura^1^, Kathryn Hooper^1^, Yuanyuan Xiao^1^, Michelle Blake^1^, Max Fiebiger^1^, Radha Dhayal^1^, Christine Saechao^1^, Alexandre Iannello^1^, Fred Ramsdell^1^, Jeffrey A. Bluestone^1^, Josh Beilke^1^

Affiliations:

^1^ Sonoma Biotherapeutics, South San Francisco, CA and Seattle, WA, USA

^2^ Aclairo Pharmaceutical Development Group, Vienna, VA

Corresponding Author: Herve Lebrec

Keywords: Regulatory T cells, Chimeric Antigen Receptor, Immunosuppression, New Approach Methodologies

**Supplementary Material**

# Supplemental Tables

**Supplemental Table 1. Confirmed Off-Target Binding of the SBBT777101 scFv in the Retrogenix Cell Microarray Platform**

| **Target Name** | **Accession** | **Library Screen (Fixed Cells)** | | **Confirmation Screen (Fixed Cells)** | | **Confirmation Screen (Live Cells)** | **Flow Cytometry** | | | |
| --- | --- | --- | --- | --- | --- | --- | --- | --- | --- | --- |
|  |  | **Rep 1** | **Rep 2** | **Rep 1** | **Rep 2** |  | **7101-huIgG1-Fc** | **7101-huIgG1** | **Isotype-huIgG1** | **7101-mIgG2a-Fc** |
| Citrullinated Fibrinogen | n/a | Strong | Strong | Strong | Strong | NT | NT | NT | NT | NT |
| Citrullinated Vimentin | n/a | Strong | Strong | Strong | Strong | NT | NT | NT | NT | NT |
| FCGR3B | NM_000570.3 | Medium | Weak/med | Weak/med | Weak/med | Weak/med | Strong | Strong | Strong | None |
| FCGR3A | BC017865.1 | Weak/med | Weak | Weak | Weak | Weak/med | Strong | Strong | Strong | None |
| FCGR2B | NM_001002273.2 | Medium | Medium | Weak/medium | Weak | Medium | Strong | Strong | Strong | None |

7101-huIgG1-Fc=SBT777101 single-chain variable fragment (scFv) fused to a human IgG1 Fc region; 7101-huIgG1=SBT777101 scFv full-length human IgG1 antibody; 7101-mIgG2a=SBT777101 scFv fused to a mouse IgG2a Fc region; FCGR = Fc Gamma Receptor; med = medium; n/a = not applicable; Isotype-huIgG1=non-targeting control scFv full-length human IgG1 antibody; NT = not tested; Rep = replicate

**Supplemental Table 2. Mouse Single Dose Toxicity Study Pathology; Day 14**

|  | | Male | | | | Female | | | |
| --- | --- | --- | --- | --- | --- | --- | --- | --- | --- |
| Group Number | | 1 | 2 | 3 | 4 | 1 | 2 | 3 | 4 |
| No. Animals/group | | 5 | 5 | 5 | 5 | 5 | 5 | 5 | 5 |
| **Kidneys**  Basophilic tubules | Number Examined | 5 | 5 | 5 | 5 | 5 | 5 | 5 | 5 |
|  | Unremarkable | 5 | 5 | 4 | 5 | 5 | 5 | 5 | 5 |
|  |  | - | - | 1 | - | - | - | - | - |
|  | Grade 1 | - | - | 1 | - | - | - | - | - |
| **Liver**  Necrosis | Number Examined | 5 | 5 | 5 | 5 | 5 | 5 | 5 | 5 |
|  | Unremarkable | 5 | 5 | 5 | 5 | 5 | 5 | 5 | 4 |
|  |  | - | - | - | - | - | - | - | 1 |
|  | Grade 1 | - | - | - | - | - | - | - | 1 |
| **Lung**  Alveolar macrophages, increased  Ectasia  Thrombus | Number Examined | 5 | 5 | 5 | 5 | 5 | 5 | 5 | 5 |
|  | Unremarkable | 3 | 1 | 1 | 1 | 2 | 2 | 1 | 1 |
|  |  | 1 | 1 | 1 | 3 | 1 | 2 | 3 | 4 |
|  | Grade 1 | 1 | 1 | 1 | 3 | 1 | 2 | 3 | 4 |
|  |  | 1 | 3 | 3 | 1 | 3 | 2 | 2 | 2 |
|  | Grade 2 | 1 | 3 | 2 | - | 2 | 1 | 2 | 2 |
|  | Grade 3 | - | - | 1 | 1 | 1 | 1 | - | - |
|  |  | - | - | - | 1 | - | - | - | 1 |
|  | Grade 1 | - | - | - | 1 | - | - | - | - |
| **Site, Injection**  Infiltrate, mononuclear cell  Pustule | Number Examined | 5 | 5 | 5 | 5 | 5 | 5 | 5 | 5 |
|  | Unremarkable | 4 | 5 | 4 | 5 | 5 | 5 | 5 | 5 |
|  |  | 1 | - | - | - | - | - | - | - |
|  | Grade 2 | 1 | - | - | - | - | - | - | - |
|  |  | - | - | 1 | - | - | - | - | - |
|  | Grade 1 | - | - | 1 | - | - | - | - | - |
| **Spleen**  Extramedullary hematopoiesis | Number Examined | 5 | 5 | 5 | 5 | 5 | 5 | 5 | 5 |
|  | Unremarkable | 0 | 0 | 0 | 0 | 0 | 0 | 0 | 0 |
|  |  | 5 | 5 | 5 | 5 | 5 | 5 | 5 | 5 |
|  | Grade 1 | 2 | - | - | - | 1 | - | 1 | - |
|  | Grade 2 | 2 | 4 | 5 | 3 | 3 | 1 | 4 | - |
|  | Grade 3 | 1 | 1 | - | 2 | 1 | 4 | - | 5 |
| **Testes**  Degeneration  Multinucleated giant cell  Necrosis | Number Examined | 5 | 5 | 5 | 5 | - | - | - | - |
|  | Unremarkable | 3 | 4 | 5 | 4 | - | - | - | - |
|  |  | - | - | - | 1 | - | - | - | - |
|  | Grade 1 | - | - | - | 1 | - | - | - | - |
|  |  | 2 | - | - | - | - | - | - | - |
|  | Grade 1 | 2 | - | - | - | - | - | - | - |
|  |  | - | 1 | - | - | - | - | - | - |
|  | Grade 2 | - | 1 | - | - | - | - | - | - |
| **Thymus**  Cyst | Number Examined | 5 | 5 | 4 | 3 | 4 | 4 | 5 | 5 |
|  | Unremarkable | 0 | 2 | 2 | 1 | 2 | 3 | 3 | 1 |
|  |  | 5 | 3 | 2 | 2 | 2 | 1 | 2 | 4 |
|  | Grade 1 | 1 | 1 | - | - | - | 1 | - | 3 |
|  | Grade 2 | 4 | 2 | 2 | 2 | 2 | - | 2 | 1 |
| **Glands, preputial**  Cyst | Number Examined | - | - | - | 1 | - | - | - | - |
|  | Unremarkable | - | - | - | 0 | - | - | - | - |
|  |  | - | - | - | 1 | - | - | - | - |
|  | Grade 3 | - | - | - | 1 | - | - | - | - |

No. = number

**Supplemental Table 3. Tissue Binding Profile of SBT777101 scFv in NCG Mouse Tissues Following Treatment with LPS and/or PBMC**

| **Tissue** | **Cell Type** | **Control** | **Irradiation Only** | **LPS Only** | **Irradiation + PBMC + LPS** | **Irradiation + PBMC** |
| --- | --- | --- | --- | --- | --- | --- |
| Kidney | Extracellular Material | **++** | **+++** | **++** | **++** | **++** |
|  | Mononuclear Leukocytes | **+++** | **+++** | **++** | **+** | **+** |
|  | Epithelial cells (tubules) | **+++** | **++** | **++** | **++** | **+++** |
| Brain | Epithelial cells (ependyma) | **++** | **++** | **+++** | **+++** | **+++** |
| Liver | Extracellular material | **++** | **+** | **+** | **++** | **++** |
| Spleen | Mononuclear leukocytes | **-** | **+** | **+** | **+** | **-** |
| Lung | Extracellular material | **+** | **+** | **++** | **+++** | **++** |
|  | Mononuclear leukocytes | **+** | **+** | **+** | **++** | **++** |
|  | Endothelial cells | **-** | **-** | **+** | **+** | **+** |
|  | Epithelial cells | **-** | **-** | **+** | **+** | **+** |
| Large Intestine | Extracellular material | **+** | **+** | **++** | **++** | **+** |

Staining was assessed in frozen tissues collected from mice at Days 3, 7, and 10 (n=5 per time point; n=15 per group). Only tissues and staining patterns with observed changes relative to control are shown. Symbols indicate the number of animals with positive staining at any time point: - = 0 animals; + = 1-5 animals; ++ = 6-10 animals; +++ = 11-15 animals.

# Supplemental Figures

**Supplemental Figure 1. Detection of CAR Vector Sequences in Tissues by ddPCR Following Single-Dose Administration in NCG Mice.** CAR vector sequences (RRE copies/reaction) were measured by ddPCR in blood, brain, liver, lung, and spleen collected at Day 7 (top) and Day 14 (bottom) necropsy following single dose administration of CAR^-^/EGFR^+^ Tregs, SBT777101, or vehicle. Dashed lines indicate the limit of blank (LOB) and lower limit of quantitation (LLOQ). Gray dots represent samples below the LOB; blue dots represent samples above the LOB but below the LLOQ.
